# Engineered probiotic restores GLP-1 signaling to ameliorate fiber-deficiency exacerbated colitis

**DOI:** 10.1126/sciadv.adx6869

**Published:** 2025-11-07

**Authors:** Leonie Brockmann, Carlotta Ronda, Logan T. Schwanz, Yiming Qu, Daniel W. Shneider, Chrystal F. Mavros, Ivaylo I. Ivanov, Govind Bhagat, Harris H. Wang

**Affiliations:** ^1^ Department of Systems Biology, Vagelos College of Physicians and Surgeons, Columbia University, New York, NY 10032, USA.; ^2^ Human Biology-Microbiome-Quantum Research Center (WPI-Bio2Q), Keio University, Tokyo 108-8345, Japan.; ^3^ Innovative Genomics Institute at UC Berkley, Berkley, CA 94720, USA.; ^4^ Integrated Program in Cellular, Molecular, and Biomedical Studies, Columbia University, New York, NY 10032, USA.; ^5^ Department of Genetics and Development, Columbia University Medical Center, New York, NY 10032, USA.; ^6^ Department of Microbiology and Immunology, Vagelos College of Physicians and Surgeons, Columbia University, New York, NY 10032, USA.; ^7^ Department of Pathology and Cell Biology, Vagelos College of Physicians and Surgeons, Columbia University, New York, NY 10032, USA.; ^8^ Department of Biomedical Engineering, Fu Foundation School of Engineering and Applied Sciences, Columbia University, New York, NY 10027, USA.; ^9^ Columbia University Digestive and Liver Disease Research Center, New York, NY 10032, USA.

## Abstract

Western diets reduced in fiber promote dysbiosis and exacerbate colitis, with short-chain fatty acids (SCFAs) from fiber fermentation known to regulate glucagon-like peptide 1 (GLP-1) secretion. Whether GLP-1 dysregulation directly links diet-induced dysbiosis to colitis severity, and if this pathway can be therapeutically targeted independently of dietary fiber, remains unclear. Here, we show that dextran sulfate sodium (DSS)–induced colitis severity correlates with compensatory GLP-1 increases, while receptor blockade worsens damage, confirming GLP-1’s protective role during colitis. Fiber deficiency impaired L cell function and GLP-1 release, increasing colitis susceptibility. GLP-1 receptor agonist reversed these effects, restoring barrier integrity and accelerating recovery. We engineered a probiotic, releasing a microbial peptide, that locally elevates GLP-1, which normalized gut parameters in fiber-deprived mice and alleviated colitis via GLP-1–dependent mechanisms, including improved metabolism, antimicrobial defenses , and barrier restoration. Our findings mechanistically connect fiber deficiency to colitis through GLP-1 and demonstrate that probiotic-mediated GLP-1 modulation can bypass dietary fiber requirements to maintain gut homeostasis.

## Teaser

Engineered probiotic delivers bacterial peptide that enhances GLP-1 and restores barrier integrity in diet-modulated colitis.

## INTRODUCTION

Gastrointestinal dysfunction and dysbiosis are increasingly prevalent concerns in westernized societies, where modern dietary habits emphasize processed foods low in fiber. These diets disrupt the gut microbiota, impair production of microbial metabolites, and alter metabolic signaling in intestinal cells (*1*–*4*), ultimately driving local and systemic inflammatory responses (*5*–*8*). While fiber deficiency is known to exacerbate colitis in mouse models (*9*–*11*), and fiber has been demonstrated to influence enteroendocrine L cells and hormonal glucagon-like peptide 1 (GLP-1) levels (*12*–*14*), the mechanistic relationship between diet-induced L cell dysfunction and colitis severity remains poorly understood.

The gut’s local endocrine system is instrumental in maintaining both intestinal homeostasis and systemic health. Among enteroendocrine cell (EEC) subtypes, L cells residing in the ileal and colonic epithelium, have garnered particular attention, since their secreted peptide hormones, especially GLP-1, have emerged as potential modulators of inflammation (*15*–*18*). Although alterations in this endocrine system have been noted in patients with inflammatory bowel disease (IBD) (*19*, *20*), the interplay between the microbiota and EECs in IBD pathophysiology remains mechanistically unresolved. Notably, circulating GLP-1 levels are chronically elevated in patients with IBD (*19*). While GLP-1 is best known for its roles in regulating food intake, gastric emptying, and insulin secretion (*21*), its receptors extend beyond pancreatic β cells to include some gut epithelial cells and a range of immune cells (*17*, *22*–*24*), suggesting its involvement in modulating gut-immune interactions. However, despite these advances, mechanistic insights into GLP-1’s role in colitis and intestinal metabolic injury remain incomplete.

Dietary fiber, which is metabolized into short-chain fatty acids (SCFAs) by the gut microbiome, is a modulator of L cell biology and a primary energy source for colonic cells (*12*, *25*). Despite the well-known benefits of adequate fiber intake, many individuals in western countries fall short of recommended daily amounts (*26*). Moreover, patients with active IBD often adopt low-fiber diets to alleviate symptoms (*27*), potentially exacerbating dysbiosis. Consequently, there is a need for novel therapeutic approaches beyond dietary modifications alone to address colitis-associated dysbiosis and reinforce gut barrier function. Recent research demonstrated that bacterial-derived peptides can also modulate enteroendocrine responses. In a previous screen of bacterial products that can stimulate gut peptide hormone release, the δ-toxin (Hld_SE_), a 25–amino acid peptide (MAADIISTIGDLVKWIIDTVNKFKK) from commensal *Staphylococcus epidermidis*, was shown to induce GLP-1 release in vitro (*28*), thus making it an intriguing candidate for engineered probiotic solutions.

Our study reveals how fiber deficiency exacerbates colitis through impaired L cell function and reduced GLP-1 production, establishing a previously unrecognized mechanistic link between diet and disease progression. We further demonstrate that pharmacological blockade of endogenous GLP-1 signaling worsens dextran sulfate sodium (DSS) colitis, directly confirming its essential protective role in intestinal inflammation. Most significantly, we have developed an engineered probiotic (*EcN-Hld_SE_*) that addresses the limitations of dietary interventions by locally restoring GLP-1 signaling independent of fiber intake, thereby effectively mitigating colitis manifestations. These findings provide insights into how dietary factors influence colitis through the gut endocrine system while introducing a novel therapeutic strategy that leverages host-microbe interactions to restore gut homeostasis.

## RESULTS

### GLP-1 is a mediator of mucosal defense against intestinal injury and bacterial invasion

**Fig. 1.**
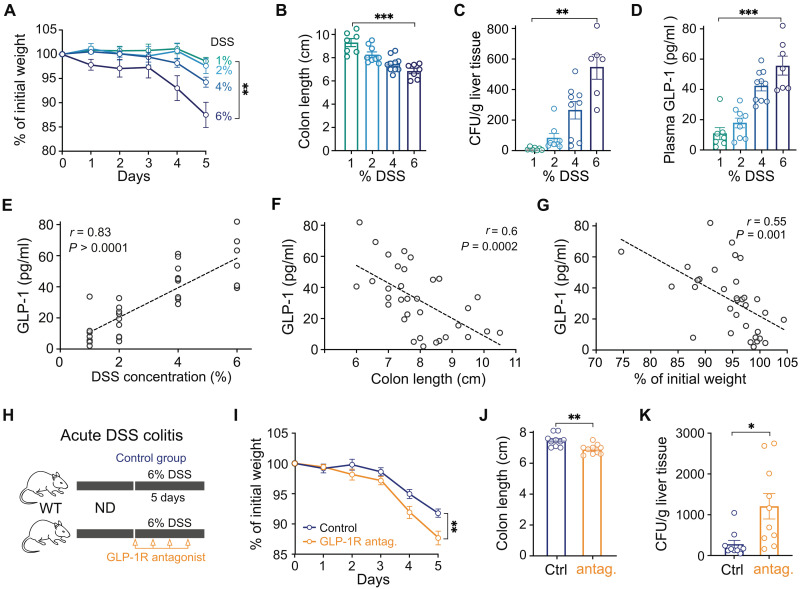
Dose-dependent DSS colitis severity correlates with rising GLP-1 levels, while GLP-1 receptor blockade exacerbates colitis symptoms. (**A**) Weight development of ND mice during 5 days of DSS colitis with indicated DSS concentrations. Cumulative of three independent experiments (*n* = 7 to 10 mice per group). (**B**) Colon length of ND mice after 5 days of DSS colitis with indicated DSS concentrations. Cumulative of three independent experiments (*n* = 7 to 10 mice per group). (**C**) CFU in the liver of ND mice after 5 days of DSS colitis with indicated DSS concentrations. Cumulative of three independent experiments (*n* = 7 to 10 mice per group). (**D**) Concentration of GLP-1 in plasma of ND mice after 5 days of DSS colitis with indicated DSS concentrations. Cumulative of three independent experiments (*n* = 7 to 10 mice per group). (**E**) Correlation between plasma GLP-1 and DSS concentration of ND mice after 5 days of DSS colitis. Cumulative of three independent experiments (*n* = 33 mice). (**F**) Correlation between plasma GLP-1 and colon length of ND mice after 5 days of DSS colitis. Cumulative of three independent experiments (*n* = 33 mice). (**G**) Correlation between plasma GLP-1 and % of initial weight of ND mice after 5 days of DSS colitis. Cumulative of three independent experiments (*n* = 33 mice). (**H**) Experimental schematic of ND mice under 6% DSS colitis condition with additional treatment with GLP-1 receptor antagonist (Exendin-9-39). (**I**) Weight development of ND mice during 5 days of DSS colitis with or without treatment with GLP-1 receptor antagonist. Cumulative of two independent experiments (*n* = 10 mice per group). (**J**) Colon length of ND mice after 5 days of DSS colitis with or without treatment with GLP-1 receptor antagonist. Cumulative of two independent experiments (*n* = 10 mice per group). (**K**) CFU in the liver of ND mice after 5 days of DSS colitis with or without treatment with GLP-1 receptor antagonist. Cumulative of two independent experiments (*n* = 10 mice per group).

In the context of gut inflammation, L cell–derived GLP-1 is released in response to intestinal damage in a Toll-like receptor 4 (TLR4)–dependent manner (*17*, *29*). This observation suggests a potential role of GLP-1 in intestinal mucosal defense. To examine this, we first assessed GLP-1 responses after intestinal damage using the acute DSS colitis model, which causes epithelial injury (*30*). As expected, increasing DSS concentrations worsened colitis severity, as shown by increased weight loss and colon shortening, and increasing bacterial translocation from the gut into the liver (Fig. 1, A to C). Circulating GLP-1 levels correspondingly rose with increased colitis severity (Fig. 1D). The level of GLP-1 showed strong correlation with DSS concentration (Fig. 1E) and parameters of disease activity, such as colon length and weight loss (Fig. 1, F and G), indicating its potential role as both a biomarker of intestinal health and a functionally relevant mediator in disease pathology. To directly test the role of GLP-1 in gut inflammation, we blocked GLP-1 receptor during high-dose DSS colitis (6% DSS), a condition in which we observed a significant rise in plasma GLP-1 levels, using the GLP-1 receptor antagonist Exendin-9-39 (Fig. 1H). Blocking GLP-1 signaling resulted in exacerbated disease activity, leading to more weight loss, greater colon shortening, and higher bacterial dissemination into the liver (Fig. 1, I to K). Elevated bacterial translocation suggested a further breakdown of gut barrier function in the absence of GLP-1 signaling during DSS-induced intestinal injury, thus demonstrating a crucial role of GLP-1 for maintenance of mucosal integrity. In contrast, when we applied the same approach to mice treated with only 2% DSS, we detected no worsening of colitis parameters such as colon length or weight loss, suggesting that GLP-1 signaling is not essential for protection against mild colitis, which does only induce a modest GLP-1 release (Fig. 1D and fig. S1, A to C), although we cannot exclude subtle effects on other parameters such as bacterial translocation. Notably, when we treated normal diet (ND) mice with a GLP-1 receptor agonist (Exendin-4) during 6% DSS colitis, we observed an initial drop in weight, likely due to the known anorectic effect of GLP-1 receptor activation; however, only modest improvement was seen in parameters such as colon length and bacterial translocation. This limited effect may be attributed to the already elevated levels of endogenous GLP-1 under these conditions, potentially masking additional benefits of pharmacologic GLP-1 receptor stimulation (fig. S1, D to G).

### Release of GLP-1 in response to tissue damage is dysregulated upon fiber deficiency

To investigate diet-dependent enteroendocrine function, we compared mice fed standard laboratory chow (ND) with those fed a semi-purified fiber-deficient diet (FDD) for 5 weeks (fig. S2A). While both diets had similar caloric density, we note that they differ in multiple nutritional components including ingredient sources and processing methods (see Materials and Methods). Compared to ND mice, FDD mice exhibited reduced weight gain, colon shortening, and increased mucosal lymphoid aggregates in the colon (fig. S2, B to D). Accordingly, we observed up-regulation of genes involved in immune cell activation and neutrophil migration (fig. S2E) and signs of weakened barrier function, as indicated by down-regulation of genes encoding tight junction proteins and increased infiltrating neutrophils (fig. S2, F and G).

**Fig. 2.**
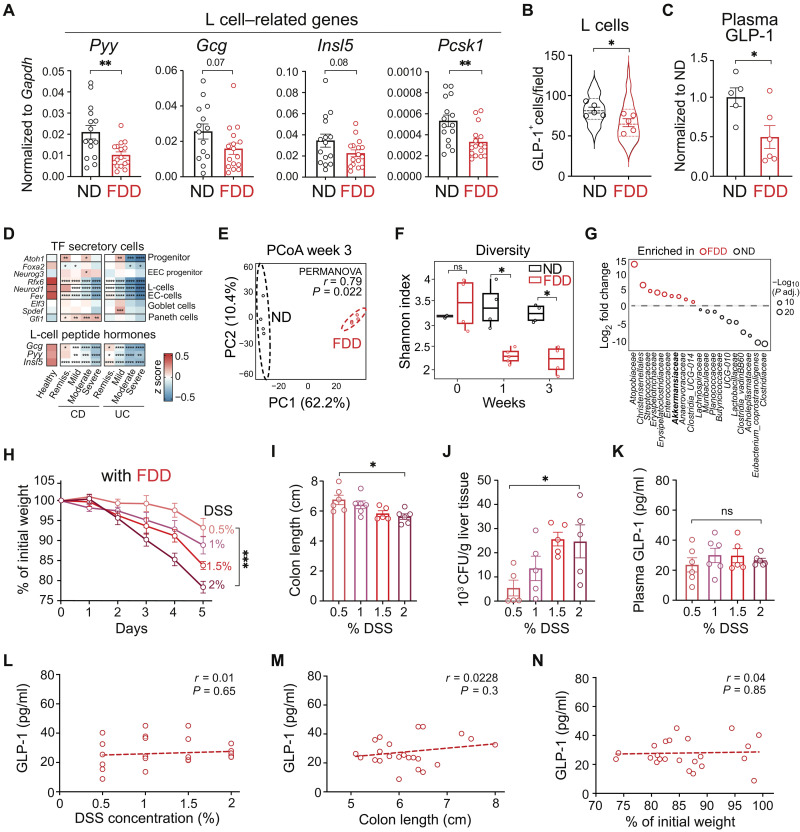
L cell biology and GLP-1 release are dysregulated during fiber deficiency–induced dysbiosis. (**A**) Quantitative polymerase chain reaction (qPCR) of selected L cell related genes from colon of ND- or FDD-fed mice (5 weeks). Cumulative of three independent experiments (*n* = 14 to 16 mice per group). (**B**) L cell numbers by GLP-1 staining in colon of ND- or FDD-fed mice (5 weeks). Data shown as average per biological replicate (*n* = 5 mice per group; 10 fields per colon), overlaid on violin plot representing all imaged fields. One experiment. (**C**) Plasma GLP-1 concentration in ND or FDD mice after 1 week. Cumulative of two independent experiments (*n* = 5 to 6 mice per group). Values normalized to ND average in each experiment. (**D**) Heatmap of selected transcription factors and L cell–related genes in human colon biopsies (*31*). One-way ANOVA versus healthy controls. (**E**) Principal coordinates analysis (PCoA) of Aitchison distances (Euclidean on CLR-transformed ASV counts) from 16*S* ribosomal RNA (rRNA) sequencing of fecal samples after 3 weeks on ND or FDD. One experiment (*n* = 4 mice per group); significance by permutational multivariate analysis of variance (PERMANOVA) (999 permutations). (**F**) Shannon diversity index of fecal microbiota at baseline and after 1 or 3 weeks on ND or FDD. Box and whisker plots show interquartile range (IQR) and 1.5× IQR. Pairwise differences assessed by two-sided Wilcoxon test. One experiment (*n* = 4 to 5 mice per group). (**G**) Family-level differential-abundance bubble plot from (**E**). Each bubble reflects a significantly changed bacterial family (Benjamini-Hochberg adjusted *P* < 0.05), plotted by log_2_ fold change and −log_10_ adjusted *P* value. (**H** to **J**) Weight loss (H), colon length (I), and liver CFUs (J) in FDD mice after 5 days of DSS colitis at indicated DSS concentrations, following 1 week of FDD feeding. Cumulative of three independent experiments (*n* = 5 to 6 mice per group). (**K** to **N**) Plasma GLP-1 levels in DSS-treated FDD mice (K; *n* = 5 to 6 mice per group), correlations with DSS concentration (L), colon length (M), and weight loss (N) (correlations *n* = 23 mice). Cumulative of three independent experiments.

Concurrently, we observed a decrease in gene expression of L cell–derived peptide hormones (*Pyy*, *Insl5*, and *Gcg*) and the proglucagon processing enzyme *Pcsk1* (encoding prohormone convertase 1/3) in the colon of FDD mice (Fig. 2A). While the reduction in *Gcg* expression showed a consistent trend but did not reach statistical significance, the marked down-regulation of *Pcsk1* suggests impaired processing of proglucagon to active GLP-1. Expression changes were accompanied by a reduced number of immunodetectable GLP-1^+^ cells in the colon (Fig. 2B and fig. S2H), which could reflect either depletion of L cells or diminished intracellular stores. Enzyme immunoassay of plasma samples revealed lower levels of circulating GLP-1 in mice fed FDD for 1 week (Fig. 2C), indicating that the transcriptional and posttranslational changes ultimately translate to reduced systemic GLP-1 availability. Moreover, the transcriptome analysis of colon biopsies from patients with IBD who often have fiber deficiency (*31*) also revealed the down-regulation of transcription factors driving enteroendocrine and L cell differentiation (*Neurod1*, *Rfx6*, and *Fev*) and L cell–derived peptide hormones (*Gcg*, *Pyy*, and *Insl5*) even during disease remission (Fig. 2D).

Diet is a strong modulator of gut microbiota composition. Hence, FDD caused a significant shift within the intestinal microbiome with a marked reduction in bacterial diversity, accompanied by a decrease in fiber-dependent bacteria from *Bacteroides*, *Lactobacillales*, and *Clostridiales* groups. We observed a decrease of many SCFA-producing bacteria (e.g., Clostridiaceae, Butyricicoccaceae, and Lachnospiraceae) and an increase in strains with nonfiber-dependent metabolisms including mucus-degrading strains such as *Akkermansia* (Fig. 2, E to G). These microbiome changes may contribute to L cell disturbances under FDD.

Building on these observations, we next explored the impact of diet-induced dysbiosis and L cell dysregulation in models of colonic injury and inflammation. In both the acute DSS and chronic *Il10*^−/−^ colitis models, FDD mice exhibited significantly worsened disease, including high mortality, severe weight loss, colon shortening, and bacterial translocation into the liver compared to ND mice (fig. S3, A to M). Diet-modulated colitis consistently correlated with severe gut barrier dysfunction. This effect was, at least partially, attributed to the absence of SCFAs, since supplementing the SCFA butyrate in drinking water of FDD mice eased DSS colitis severity (fig. S3, N to Q). Notably, while butyrate supplementation ameliorated colitis severity in FDD mice, it did not significantly alter plasma GLP-1 levels compared to unsupplemented FDD controls, likely reflecting its broader mucosal-protective effects that reduced the pathological drive for GLP-1 secretion (fig. S3R).

Given the markedly enhanced susceptibility to DSS-induced colitis and significantly higher mortality observed in FDD mice, we next assessed the range of GLP-1 responses using lower DSS concentrations. In FDD mice, GLP-1 release in response to tissue damage during DSS-induced colitis appeared dysregulated. Even at a low DSS concentration, FDD mice exhibited severe pathology, which progressively worsened with increasing DSS levels (Fig. 2, H to J). Notably, based on key indicators such as colon length, weight loss, and colony forming units (CFU) counts in the liver, all measured using DSS from the same manufacturing lot, FDD mice exposed to just 0.5% DSS displayed pathology comparable to ND mice given as much as 6% DSS. GLP-1 levels in FDD mice were already elevated at low DSS concentrations, correlating with heightened pathology (Fig. 2K). However, unlike ND mice, which demonstrated a dynamic up-regulation of GLP-1 in response to higher DSS concentrations (Fig. 1D), FDD mice failed to exhibit this response, with GLP-1 levels (Fig. 2K) plateauing below those observed in ND mice experiencing similar levels of pathology. This suggests that L cells in FDD mice are unable to further increase systemic GLP-1 levels. In accord, GLP-1 levels were not correlated with DSS concentration or disease activity in FDD mice (Fig. 2, L to N) in contrast to ND mice (Fig. 1, E to G), highlighting the dysregulation of L cell function under diet-modulated inflammatory conditions. This disruption in GLP-1 release in FDD mice could potentially contribute to the colitis severity in these mice. While GLP-1 levels are elevated in patients with IBD, they also do not correlate with disease activity (*19*), mirroring our observations in FDD mice. Combined with our finding that L cell–related genes are down-regulated in patients with IBD (Fig. 2D), this suggests potential dysregulation of the GLP-1 signaling pathway in at least a subset of patients with IBD.

### GLP-1 receptor signaling improves colitis features in dysbiotic mice

**Fig. 3.**
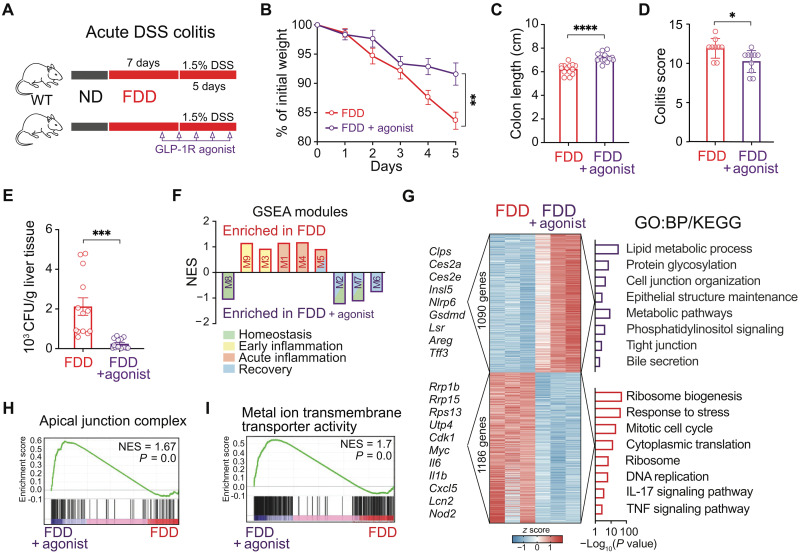
GLP-1 receptor agonist can ameliorate severe colitis in dysbiotic mice. (**A**) Experiment schematic of DSS colitis conditions with GLP-1 receptor agonist (Exendin-4) treatment in FDD mice. (**B**) Weight development of FDD mice during 5 days of DSS colitis with or without GLP-1 receptor agonist treatment. Cumulative of three independent experiments. *n* = 16 to 17 mice per group. (**C**) Colon length of FDD mice after 5 days of DSS colitis with or without GLP-1 receptor agonist treatment. Cumulative of three independent experiments. *n* = 14 mice per group. (**D**) Colitis score based on assessment of hematoxylin and eosin (H&E) staining of colon from FDD or FDD mice treated with GLP-1 receptor agonist after 5 days of DSS colitis. Two independent experiments. *n* = 10 to 11 mice per group. (**E**) CFU in the liver of FDD mice after 5 days of DSS colitis with or without GLP-1 receptor agonist treatment. Cumulative of three independent experiments. *n* = 14 mice per group. (**F**) GSEA of RNA sequencing samples from colon of FDD mice after 5 days of DSS colitis with or without GLP-1 receptor agonist treatment with modules (gene lists) identified in DSS colitis time series (*32*). One experiment (*n* = 3 mice per group). (**G**) Heatmap of differentially expressed genes (DEGs) from RNA sequencing samples from colon of FDD mice after 5 days of DSS colitis with or without GLP-1 receptor agonist treatment, top Kyoto Encyclopedia of Genes and Genomes (KEGG) and GO:Biological Process (BP) pathways in over representation analysis on the right and example DEGs on the left. One experiment (*n* = 3 mice per group). (**H**) Enriched GO:Cellular Component (CC) term apical junction complex in GSEA from RNA sequencing samples in (G) with leading edge gene expression on the right. (**I**) Enriched GO:Molecular Function (MF) term “metal ion transmembrane transporter activity” in GSEA from RNA sequencing samples in (G) with leading edge gene expression on the right.

We next assessed whether elevating GLP-1 responses could improve acute DSS colitis outcomes in FDD mice. Treatment with GLP-1 receptor agonist (Exendin-4) during DSS colitis (Fig. 3A) led to reduced weight loss and less colon shortening in FDD mice (Fig. 3, B and C). Histological colitis score also improved with GLP-1 receptor agonist treatment (Fig. 3D). Restoration of GLP-1 signaling significantly reduced CFU levels in the liver, indicating enhanced gut barrier function (Fig. 3E). To control for potential confounding effects of GLP-1 agonism on water intake, we confirmed that the protective effects persisted even when DSS was administered by oral gavage (rather than ad libitum in drinking water), ensuring consistent colitis induction across groups (fig. S4, A to D). The RNA sequencing of the colon from Exendin-4–treated FDD mice revealed a transcriptional shift during acute colitis. Using a time-series dataset of DSS colitis (*32*), we performed Gene Set Enrichment Analysis (GSEA) that revealed enrichment of gene modules associated with homeostasis and recovery phases in response to GLP-1 agonist treatment (Fig. 3F). Differential expression analysis showed GLP-1 receptor agonist up-regulated genes for lipid metabolism (e.g., *Mlxipl*, *Ces2a*, *Ces2b*, *Cyp4f40*, and *Clps*), epithelial integrity (e.g., *Ocln*, *Cldn8*, *Marveld2*, *Marveld3*, *Lsr*, and *Neurod1*), and bile secretion (e.g., *Abcc3*, *Aqp4*, and *Abcb1a*), while down-regulating ribosome biogenesis (e.g. *Rrs1*, *Rrp12*, and *Rcl1*), cell cycle (e.g. *Myc*, *Ccne1*, *Tuba8*, and *Mcm3*), and inflammation genes (e.g., *S100a8*, *Il6*, *Lcn2*, *Il1b*, and *Mmp9*) (Fig. 3G). GSEA identified enriched apical junction and ion transport pathways (Fig. 3, H and I). Leading-edge analysis furthermore highlighted up-regulated metabolic [e.g., tricarboxylic acid (TCA) cycle and pyruvate metabolism] and secretory (vesicle trafficking and exocytosis) processes critical for intestinal function and epithelial repair (fig. S4, E and F). Consistent with this, GLP-1 agonist treatment increased expression of L cell hormones (*Gcg*, *Pyy*, and *Insl5*), prohormone convertase (*Pcsk1*), mucus components (*Muc2*, *Tff3*, *Clca1*, and *Fcgbp*), and secretory cell transcriptional regulators (e.g., *Atoh1*, *Foxa2*, and *Neurod1*) (fig. S4G), suggesting that GLP-1 may enhance secretory cell populations, as supported by in vitro studies (*33*). L cell numbers increased in GLP-1 receptor agonist-treated FDD mice during DSS colitis; however, mucus granules remained unchanged (fig. S4H). Overall, GLP-1 signaling enhanced the expression of genes critical for gut function, including ion and metabolite transport, metabolic pathways, and barrier integrity via apical and tight junctions. It also promoted genes involved in intestinal secretory cell differentiation and function.

### An engineered probiotic modulates GLP-1 levels and intestinal parameters in fiber-deprived mice

**Fig. 4.**
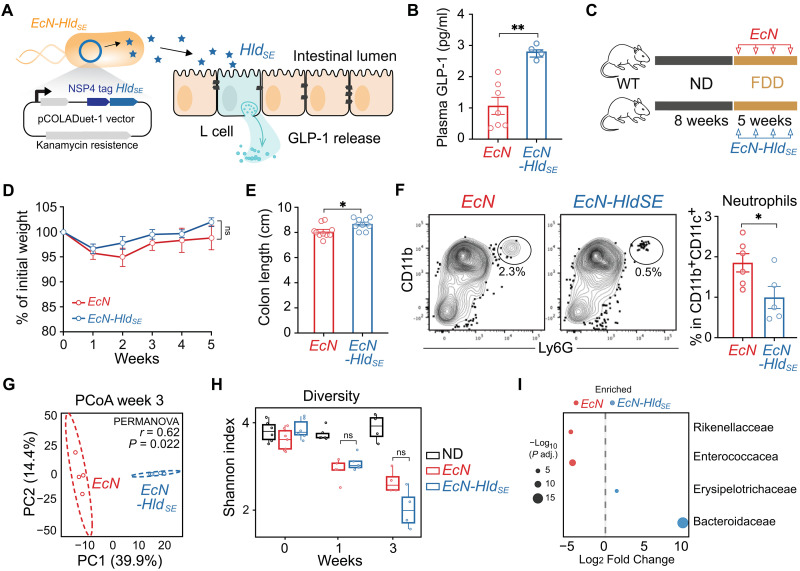
An engineered probiotic increases GLP-1 level and modulates intestinal parameters in FDD mice. (**A**) Schematic of engineered *E. coli* Nissle 1917 containing plasmid with kanamycin-resistance sequence, NSP4 secretion sequence, and Hld_SE_ peptide sequence used to engineer *EcN-Hld_SE_* (described in Materials and Methods). (**B**) Plasma GLP-1 concentrations in FDD mice following daily oral gavage of 10^10^ CFU of either *EcN* or *EcN-Hld_SE_* for 1 week. Cumulative of two independent experiments. *n* = 4 to 6 mice per group. (**C**) Experimental schematic of steady-state dietary intervention with additional gavage of 10^10^ CFU/mouse *EcN* or *EcN-Hld_SE_* every other day for 5 weeks. (**D**) Weight development during 5-week intervention described in (C). Cumulative of two independent experiment. *n* = 9 to 10 mice per group. (**E**) Colon length after feeding of FDD for 5 weeks accompanied of gavage of 10^10^ CFU/mouse *EcN* or *EcN-Hld_SE_* every other day. Cumulative of two independent experiment. *n* = 9 to 10 mice per group. (**F**) CD11b and Ly6G expression (neutrophils) in colonic myeloid cells (CD11c^+^ CD11b^+^ TCRb^−^ B220^−^) after 5-week intervention as described in (**C**). Cumulative of two independent experiments. *n* = 5 to 6 mice per group. (**G**) PCoA of Aitchison distances (Euclidean on CLR-transformed ASV counts) based on 16*S* rRNA sequencing of fecal samples after 3 weeks of FDD and probiotic treatment. Significance tested by PERMANOVA (999 permutations). *r* and *P* values shown; one experiment (*n* = 4 mice per group). (**H**) Shannon diversity index of fecal microbiota from ND or FDD mice at baseline and after 1 or 3 weeks of intervention as in (C). Boxplots show IQR and median; whiskers extend to 1.5× IQR. Pairwise comparisons by two-sided Wilcoxon test (*n* = 4 to 7 mice per group). (**I**) Family-level differential abundance plot from (G). Bubbles represent families with adjusted *P* < 0.05 (Benjamini-Hochberg), sized by −log_10_ *P* value and positioned by log_2_ fold change.

Given the established role of microbes and their metabolites in modulating endocrine function (*28*, *34*), we explored an engineered probiotic strategy to locally modulate GLP-1 levels in the intestine. Restoring endogenous GLP-1 signaling offers advantages over exogenous GLP-1 receptor agonists by leveraging natural, localized GLP-1 production, potentially minimizing systemic side effects associated with pharmacological interventions. We engineered the probiotic *Escherichia coli* Nissle 1917 (*EcN*) to produce Hld_SE_ (*EcN-Hld_SE_*) and enhanced peptide secretion using the NSP4 secretion tag (Fig. 4A) (*35*). Strikingly, FDD mice orally gavaged with *EcN-Hld_SE_* daily for 1 week showed a significant increase in circulating GLP-1 (Fig. 4B). Chronic administration of *EcN-Hld_SE_* for 5 weeks attenuated colon shortening and reduced neutrophil infiltration into the colon lamina propria, countering the effects of FDD feeding we previously observed, but did not significantly affect weight development (Fig. 4, C to F, and fig. S2, B to F). Notably, *E. coli* (containing *EcN* and *EcN-Hld_SE_* strains) were detected at comparable levels between the groups (fig. S6H). We achieved similar beneficial effects in FDD *Il10*^−/−^ mice with chronic treatment with *EcN-Hld_SE_*. FDD *Il10*^−/−^ mice showed improved weight gain, less colon shortening, and signs of improved gut barrier function, indicated by reduced bacterial translocation into the liver and increased expression of tight junction genes and reduced neutrophil level and proinflammatory CD4^+^ T cells in the colon lamina propria (fig. S5, A to H).

The engineered probiotic induced moderate shifts in the intestinal microbiota, overall bacterial diversity remained unchanged (Fig. 4, G and H). These changes were characterized by an increase in relative abundance of Erysipelotrichaceae and Bacteroidaceae, alongside a reduction in Enterococcaceae and Rikenellaceales species (Fig. 4I). This suggests that the probiotic intervention may alter diet-induced dysbiosis, potentially by restoring a microbiota composition more conducive to gut barrier integrity and immune regulation. However, no significant enrichment of major butyrate-producing strains was detected, indicating that the beneficial effects of *EcN-Hld_SE_* are not mediated by increased butyrate production.

### Dietary impact on colitis is ameliorated by engineered probiotic in a GLP-1–dependent manner

**Fig. 5.**
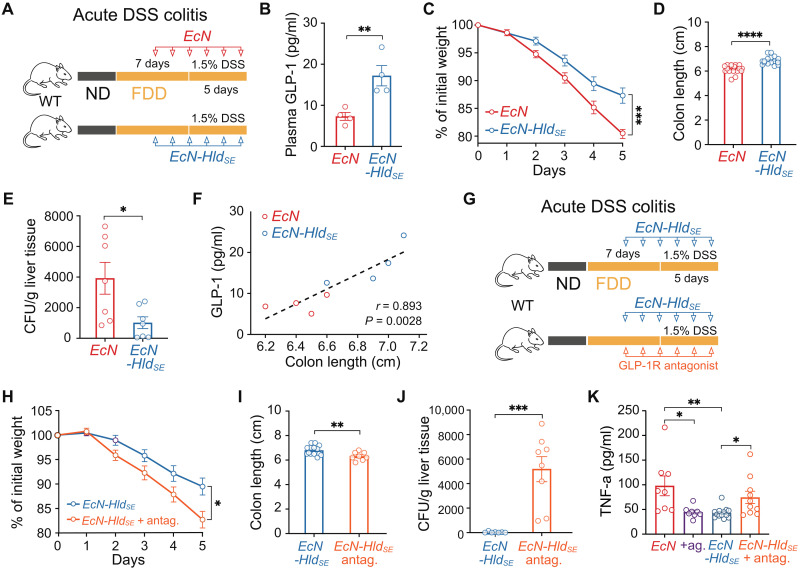
Engineered probiotic improves colitis outcomes in dysbiotic mice in a GLP-1–dependent manner. (**A**) Experiment schematic of DSS colitis with *EcN* or *EcN-Hld_SE_* treatment in FDD mice. (**B**) Plasma GLP-1 levels in FDD mice after 5 days of DSS colitis with *EcN* or *EcN-Hld_SE_* treatment. Cumulative of two independent experiments (*n* = 4 mice per group). (**C**) Weight development of FDD mice during 5 days of DSS colitis with *EcN* or *EcN-Hld_SE_* treatment. Cumulative of five independent experiments (*n* = 23 to 25 mice per group). (**D**) Colon length of FDD mice after 5 days of DSS colitis with *EcN* or *EcN-Hld_SE_* treatment. Cumulative of five independent experiments (*n* = 17 mice per group). (**E**) CFU in the liver of FDD mice after 5 days of DSS colitis with *EcN* or *EcN-Hld_SE_* treatment. Cumulative of two independent experiments (*n* = 7 mice per group). (**F**) Correlation between plasma GLP-1 and colon length of FDD mice after 5 days of DSS colitis with *EcN* or *EcN-Hld_SE_* treatment. Cumulative of two independent experiments (*n* = 8 mice per group). (**G**) Experiment schematic of DSS colitis with *EcN-Hld_SE_* treatment with or without injection of GLP-1 receptor antagonist (Exendin-9-39) in FDD mice. (**H**) Weight development during 5 days of DSS colitis as described in (G). Cumulative of three independent experiments (*n* = 11 to 15 mice per group). (**I**) Colon length of FDD mice after 5 days of DSS colitis with *EcN-Hld_SE_* treatment with or without injection of GLP-1 receptor antagonist. Cumulative of three independent experiments (*n* = 8 to 14 mice per group). (**J**) CFU in the liver of FDD mice after 5 days of DSS colitis with *EcN-Hld_SE_* treatment with or without injection of GLP-1 receptor antagonist. Cumulative of two independent experiments (*n* = 7 to 8 mice per group). (**K**) Plasma TNF-α of FDD mice after 5 days of DSS colitis with either *EcN*, *EcN* + GLP-1 receptor agonist, *EcN-Hld_SE_*, or *EcN-Hld_SE_* + GLP-1 receptor antagonist treatment. Cumulative of three independent experiments (*n* = 8 to 13 mice per group).

During acute DSS colitis, the daily treatment of FDD mice with *EcN-Hld_SE_* also significantly increased circulating GLP-1 (Fig. 5, A and B) and reduced weight loss and colon shortening (Fig. 5, C and D). *EcN-Hld_SE_* decreased bacterial dissemination into the liver, suggesting improved gut barrier function (Fig. 5E). A positive correlation between colon length and GLP-1 levels further suggested that GLP-1 restoration played a key role in these protective effects (Fig. 5F). Notably, the therapeutic benefit of *EcN-Hld_SE_* was dose dependent (fig. S6, A to C). In addition, direct comparison with an isogenic *EcN* strain (*EcN-plasmid*) carrying the empty plasmid (fig. S6, D to G) confirmed that the protective effects on weight loss, colon length, and bacterial translocation were specific to Hld_SE_ expression, as the plasmid-only control failed to improve these outcomes. Furthermore, neither strain (*EcN-plasmid* and *EcN-Hld_SE_*) showed changes to growth behavior compared to the original *EcN* strain (fig. S6I).

To further confirm the functional dependency of *EcN-Hld_SE_* treatment on GLP-1 signaling, we blocked GLP-1 receptor using a GLP-1 receptor antagonist (Exendin-9-39) (Fig. 5G). GLP-1 receptor antagonism abolished the protective effects of the engineered probiotic. Mice showed severe weight loss and shorter colons (Fig. 5, H and I) and notably increased number of bacteria in the liver (Fig. 5J). In addition, reduction, due to treatment with either GLP-1 receptor agonist (Exendin-4) or *EcN-Hld_SE_*, of circulating proinflammatory cytokine tumor necrosis factor–α (TNF-α) was abolished when GLP-1 receptor signaling was blocked (Fig. 5K). These results demonstrate that *EcN-Hld_SE_* induces GLP-1 release and mediates protective effects during colitis through GLP-1 receptor signaling. Notably, GLP-1 receptor antagonism did not exacerbate colitis severity in *EcN*-treated FDD mice. While GLP-1 receptor antagonism modestly increased bacterial translocation to the liver, it had no significant impact on other measures of disease activity (fig. S6, J to M). Overall, our findings underscore the potential of engineered probiotics such as *EcN-Hld_SE_* as a targeted therapeutic strategy for colitis.

**Fig. 6.**
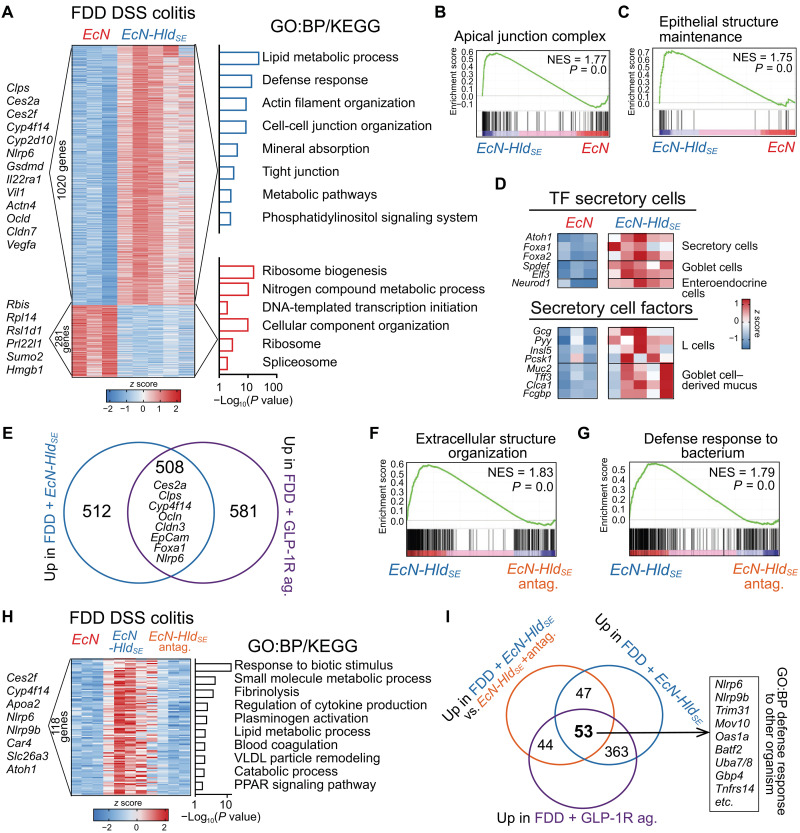
Engineered probiotic induces colon transcriptional reprogramming to enhance microbial defense pathways. (**A**) Heatmap of DEGs from colon RNA-sequencing samples of FDD mice after 5 days of DSS colitis with *EcN* or *EcN-Hld_SE_* treatment, top KEGG and GO:BP pathways from overrepresentation analysis on the right and example DEGs on the left. One experiment (*n* = 3 to 5 mice per group. (**B**) Enriched GO:CC term apical junction complex in GSEA from RNA sequencing samples in (A). (**C**) Enriched GO:BP term “epithelial structure maintenance” in GSEA from RNA sequencing samples in (A). (**D**) Heatmap of selected up-regulated transcription factors and up-regulated secretory cell linage related genes from RNA sequencing samples in (A). (**E**) Venn diagram of up-regulated DEGs from RNA sequencing comparisons: (i) FDD DSS-colitis mice with *EcN* + GLP-1 agonist versus *EcN* alone (control); (ii) FDD DSS-colitis mice with *EcN-Hld_SE_* versus *EcN* alone (control). (**F**) Enriched GO:BP term “extracellular structure organization” in GSEA from RNA sequencing of FDD mice after 5 days of DSS colitis with *EcN-Hld_SE_* treatment and with or without treatment with GLP-1 receptor agonist. One experiment (*n* = 3 to 5 mice per group). (**G**) Enriched GO:BP term “defense response to bacterium” in GSEA from RNA sequencing of FDD mice after 5 days of DSS colitis with *EcN-Hld_SE_* treatment and with or without treatment with GLP-1 receptor agonist. One experiment (*n* = 3 to 5 mice per group). (**H**) Heatmap of conserved up-regulated DEGs shared between two comparisons: (i) *EcN-Hld_SE_* versus *EcN* and (ii) *EcN-Hld_SE_* versus *EcN-Hld_SE_* + GLP-1 receptor antagonist in FDD/DSS-colitis mice. Annotated with top enriched KEGG/GO:BP pathways (right) and representative genes (left), revealing *EcN-Hld_SE_*–rescued pathways suppressed by GLP-1 receptor blockade. One experiment (*n* = 3 to 5 mice per group). (**I**) Venn diagram of up-regulated DEGs from three comparisons in FDD/DSS-colitis mice: (i) *EcN* + GLP-1 receptor agonist versus *EcN*, (ii) *EcN-Hld_SE_* versus *EcN*, and (iii) *EcN-Hld_SE_* versus *EcN-Hld_SE_* + GLP-1 receptor antagonist. Example genes on the right, enriched in GO:BP defense response to other organism.

Furthermore, the transcriptomic analysis of colon tissues from *EcN-Hld_SE_*–treated FDD mice during DSS colitis showed the up-regulation of genes involved in lipid metabolism (e.g., *Clps*, *Apoa5*, *Ces2a*, *Ces2f*, and *Cyp4f40*), tight junctions (e.g., *Marveld3*, *Ocln*, *Cldn7*, and *Tjp3*), and epithelial structure maintenance (e.g., *Lsr*, *Muc2*, *Muc13*, and *Tff3*), mirroring changes observed in mice treated with GLP-1 receptor agonist (Fig. 6A). Consistently, among the top enriched Gene Ontology (GO) terms in GSEA were “apical junction complex” and “epithelial maintenance” (Fig. 6, B and C). Overall, nearly 50% of differentially expressed genes were commonly up-regulated in the colon of FDD mice treated with *EcN-Hld_SE_* and the GLP-1 receptor agonist (Fig. 6E). These included L cell–related genes (*Gcg*, *Pyy*, *Insl5*, and *Pcsk1*), genes encoding mucus components (*Muc2*, *Tff3*, *Clca1*, and *Fcgbp*), and transcription factors driving secretory cell differentiation in the intestine (e.g., *Atoh1*, *Foxa2*, and *Neurod1*) (Fig. 6D). The transcriptional profiling of GLP-1 receptor blockade in *EcN-Hld_SE_*–treated FDD mice revealed that GLP-1 signaling was essential for maintaining gut barrier integrity and microbial defense pathways during DSS colitis. The most strongly down-regulated pathways in response to GLP-1 receptor antagonism included “extracellular structure maintenance,” highlighting GLP-1’s role in physical barrier restoration, and “defense response to bacteria,” underscoring its importance in microbial containment (Fig. 6, F and G). Comparative analysis identified 118 genes whose induction by *EcN-Hld_SE_* was critically dependent on GLP-1 receptor signaling, as evidenced by their significant down-regulation upon Exendin-9-39 treatment or in comparison to *EcN*-treated FDD mice (Fig. 6H). This cohort prominently featured genes associated with epithelial restoration (including the pH regulator *Car4* and chloride channel activators *Clca4a/b*), inflammasome function (*Nlrp6* and *Nlrc5*), and fibrinolytic processes (*Fga*, *Fgg*, and *Serpinf2*). Also represented were metabolic transporters (*Slc15a1* and *Slc26a3*) and immune defense modulators (*Zbp1*, *Ido1*, and *Gbp4*). The pathway analysis of these 118 genes further illuminated the biological processes underpinning *EcN-Hld_SE_*’s protection, with the strongest enrichments in response to biotic stimulus, reflecting heightened microbial defense, and small-molecule metabolic processes and lipid metabolism underscored metabolic effects of GLP-1 signaling. This protective signature was corroborated when we took GLP-1 receptor agonism (Exendin-4) data into consideration, which showed strong up-regulation of “defense response to other organism” pathway (Fig. 6I). The coordinated suppression of these genes following GLP-1 receptor inhibition, particularly those involved in microbial defense (e.g., *Nlrp6* and *Sectm1a*) and epithelial barrier maintenance (e.g., *Car4* and *Scin*) strongly implicates them as mechanistic effectors of the probiotic-mediated GLP-1–dependent protection.

## DISCUSSION

The increasing prevalence of diseases linked to gut barrier dysfunction, such as IBD and metabolic syndrome, underscores the need to understand pathogenic mechanisms and develop effective therapies. This study demonstrates that dysbiosis disrupts L cell biology, with L cell–derived GLP-1 playing a central role in mucosal defense and barrier integrity. Fiber deficiency compromises this protective mechanism, but restoring GLP-1 signaling, through receptor agonists or engineered probiotics, significantly alleviates colitis severity. GLP-1 activation up-regulates tight junction proteins, ion channels, and metabolic enzymes, supporting barrier restoration while enhancing the differentiation and function of secretory cell lineages, including L cells and goblet cells. We show that GLP-1 levels are dynamically up-regulated in response to intestinal tissue damage, consistent with previous findings that GLP-1 release is triggered by LPS in a TLR4- and calcium-dependent manner (*29*). This highlights GLP-1’s critical role in the mucosal response to injury. Fiber deficiency impaired GLP-1 signaling, but its restoration improved colitis outcomes and enhanced gut barrier function, likely through both local and systemic effects (*36*). Physiological improvements—such as increased colon length, reduced bacterial dissemination, and transcriptional changes in the colon—point to a significant local role for GLP-1 within the intestine.

Fiber deficiency–induced dysbiosis was characterized by an increase in mucus-degrading *Akkermansia*, consistent with prior studies (*9*). While *Akkermansia* has been associated with GLP-1 induction in other contexts (*37*), its increased relative abundance in our FDD model coincided with dysregulated GLP-1 signaling, likely reflecting the loss of fiber-dependent SCFA producers that normally dominate this regulation. The quantitative analysis revealed that *Akkermansia*’s absolute abundance remained unchanged during FDD, indicating that the observed microbial shifts primarily reflect the depletion of other taxa (*38*). Fiber deficiency also directly disrupts goblet cell biology, altering the mucus layer (*38*). Germ-free *Glp1r*^−/−^ mice exhibit impaired goblet cell differentiation and severe intestinal pathology (*39*), further emphasizing the importance of GLP-1 in maintaining gut health.

In our study, restoring GLP-1 signaling during colitis in FDD mice significantly up-regulated mucus-related genes (e.g. *Muc2* and *Tff3*), underscoring its role in goblet cell function. While our bulk RNA sequencing approach cannot resolve cell type–specific effects, the concordance between these transcriptional changes and functional improvements supports their biological relevance. Although mucus-filled vacuoles in the tissue remained unchanged, GLP-1 signaling appeared essential for supporting secretory activity. GLP-1 influenced broader transcriptional programs in secretory cell differentiation, including L cell–related genes, consistent with in vitro evidence of GLP-1 promoting L cell differentiation through paracrine signaling (*33*). Beyond secretory cell lineages, GLP-1 signaling modulated factors critical for intestinal secretion, such as ion channels, which regulate transmembrane potential. This aligns with GLP-1’s established role in pancreatic β cells, where ion channel modulation is crucial for insulin secretion (*40*, *41*). Given that mucus secretion and peptide hormone release are calcium-dependent processes (*39*, *42*, *43*), GLP-1 may enhance these functions via similar mechanisms, as supported by the enrichment of pathways related to vesicle trafficking and exocytosis.

Notably, GLP-1 signaling also induced transcriptional changes in cellular metabolism, particularly lipid metabolism, TCA cycle, and pyruvate metabolism, suggesting that it helps restore energy balance in dysbiotic mice. Our RNA sequencing analysis revealed that GLP-1–dependent protection was mediated through enrichment of defense response pathways and biotic stimulus responses, rather than canonical anti-inflammatory mechanisms. This suggests that GLP-1’s protective effects in our model may operate through a novel paradigm: Rather than directly suppressing inflammation, it strengthens essential barrier protection systems against microbial threats, likely as a downstream consequence of its metabolic adaptations. The coordinated up-regulation of microbial defense genes (e.g., *Nlrp6* and *Gbp4*) alongside metabolic transporters (e.g., *Slc15a1* and *Slc26a3*) supports this interpretation, indicating that GLP-1 helps recalibrate the gut’s equilibrium with its microbial environment under dysbiotic conditions. Our observed enrichment of Erysipelotrichaceae and Bacteroidaceae in response to the engineered probiotic may also reflect improved intestinal metabolic conditions, as these families exhibit metabolic flexibility and thrive on alternative energy sources. In contrast, fiber-dependent taxa same as many Clostridiales and *Lactobacillus* species, unaffected by the engineered probiotic, are less adaptable to fiber-deficient environments. Our findings highlight GLP-1 as a key regulator of gut barrier integrity and mucosal defense, particularly under dysbiotic conditions. Restoring GLP-1 signaling offers a potential strategy to counteract the detrimental effects of fiber deficiency and could inform innovative treatments for gut barrier dysfunction. Emerging clinical evidence further supports the translational relevance of our findings. GLP-1 receptor agonists and dipeptidyl peptidase 4 inhibitors have been used for many years in the treatment of type 2 diabetes and metabolic disorders. A recent retrospective study (*44*) reported that patients with coexisting IBD and type 2 diabetes treated with GLP-1–based therapies exhibited reduced adverse symptoms associated with IBD. While encouraging, available studies remain retrospective and do not directly assess IBD incidence or progression as primary outcomes. Thus, prospective clinical studies will be required to determine whether GLP-1–modulating therapies can provide protective benefits in patients with IBD, as predicted by our preclinical data.

The identification of the peptide Hld_SE_, derived from a skin commensal bacterium, as a potent inducer of GLP-1 in vitro, highlights the complexity of host-microbiota interactions (*28*). Although L cells are absent in the skin, this finding reveals the potential of microbial-derived molecules to influence intestinal hormone levels. Our use of an engineered probiotic to locally release Hld_SE_ and stimulate endogenous GLP-1 production exemplifies how natural processes can be harnessed for therapeutic benefit. Engineered probiotics offer a precise, localized alternative to systemic GLP-1 agonists, minimizing off-target effects (*45*). This proof-of-concept study demonstrates the feasibility of microbiome-mediated GLP-1 modulation using the *Staphylococcus*-derived δ-toxin scaffold. Comprehensive safety assessments through rigorous initial clinical trials will be required before any progression toward therapeutic use, ensuring both safety and efficacy for potential human applications. Future therapeutic development should additionally focus on identifying safer peptide alternatives, potentially derived from intestinal commensals or engineered through rational design, that maintain GLP-1–stimulating activity while eliminating potential virulence factors. The intestinal microbiota represents a particularly promising source for these bioactive molecules, given their evolutionary adaptation to host signaling pathways and mucosal environments. Understanding how specific microbes and metabolites influence GLP-1 levels could lead to novel therapies that modulate endogenous hormone production, leveraging the coevolutionary relationship between microbiota and host. In conclusion, our study demonstrates the critical role of GLP-1 in maintaining gut health and highlights the potential of engineered probiotics as a targeted therapeutic strategy for IBD and other conditions characterized by gut barrier dysfunction.

## MATERIALS AND METHODS

### Experimental design

#### Mice and diets

C57BL/6J and *Il10*^−/−^ mice were purchased from the Jackson Laboratory. Animals were purchased from maximum barrier rooms and maintained in high barrier animal rooms at Columbia University. All mice were maintained under specific pathogen–free (SPF) conditions for the duration of the experiments. To control for microbiota and cage effects, mice were shuffled between cages before the start of each experiment and randomly assigned to each group. All experiments were performed with gender and age (8 to 12 weeks at start of experiment) matched mice. Animals were maintained on ND (PicoLab Rodent Diet 20, 5053) until the start of experiments and then switched to FDD (Teklad Custom Diet, TD.00278) or continued on ND as indicated. ND contained 21% protein, 53.4% carbohydrates, 5% fat, 5% fiber, and 4.1 kcal/g. FDD contained 19.3% protein, 64.8% carbohydrates, 5.1% fat, and no fiber; overall, the diet provides 3.9 kcal/g. The most significant nutritional distinction between these diets was the absolute absence of fiber in the FDD formulation. However, secondary differences were also noted, including elevated polyunsaturated fatty acid content and variations in micronutrient composition in the FDD, as detailed in Table 1. These compositional differences may collectively influence gut microbial ecology, mucosal barrier function, and systemic immune responses in distinct ways.

**Table 1.** Nutrient composition of FDD (TD.00278) and control diet (PicoLab 20).

Category	Component	TD.00278 (FDD)	PicoLab 20 (ND)
Macronutrients	Protein (g/kg)	193	200–210
	Carbohydrates (g/kg)	648	534 (NFE) + 32.5 sucrose
	Fat (g/kg)	51	50–63
	Crude Fiber (g/kg)	0	46–60
	Energy Density (kcal/g)	3.8	4.11
Minerals	Calcium (g/kg)	6.4	6.4
	Phosphorus (g/kg)	3.6	6.4 (3.4 non-phytate)
	Potassium (g/kg)	6.2	Not listed
	Sodium (g/kg)	2.3	–
	Magnesium (g/kg)	0.5	2.2
	Iron (mg/kg)	36.5	Not specified
	Zinc (mg/kg)	35.6	–
	Copper (mg/kg)	6.0	–
	Manganese (mg/kg)	10.5	–
	Iodine (mg/kg)	0.21	–
	Selenium (mg/kg)	0.15	–
Vitamins	Vitamin A (IU/kg)	6,000	– (vitamin A acetate)
	Vitamin D (IU/kg)	1,500	– (cholecalciferol)
	Vitamin E (IU/kg)	113	– (dl-α-tocopheryl acetate)
	Vitamin K (mg/kg)	1.1	– (menadione DMPB)
	Thiamin (B1, mg/kg)	7	– (thiamine mononitrate)
	Riboflavin (B2, mg/kg)	9	–
	Niacin (mg/kg)	45	– (nicotinic acid)
	Pantothenate (B5, mg/kg)	22	– (calcium pantothenate)
	Pyridoxine (B6, mg/kg)	8.6	– (pyridoxine hydrochloride)
	Folate (mg/kg)	3	– (Folic acid)
	Vitamin B12 (mg/kg)	0.04	– (B12 supplement)
	Choline (mg/kg)	1,377	– (choline chloride)
Fatty acids and cholesterol	Saturated FA (g/kg)	7.9	7.8
	Palmitic (16:0, g/kg)	5.5	–
	Stearic (18:0, g/kg)	2.0	–
	Monounsaturated FA (g/kg)	11.5	9.6
	Oleic (18:1, g/kg)	11.5	–
	Polyunsaturated FA (g/kg)	30.5	–
	Linoleic (18:2, g/kg)	26.5	21.2
	Linolenic (18:3, g/kg)	4.0	2.7
	Cholesterol (mg/kg)	36.9	14.2

#### Animal experiment approval

All animal experiments were approved by the Institutional Animal Care and Use Committee (IACUC) of Columbia University and conducted in accordance with relevant guidelines and regulations. The study was performed under animal protocol license number AABA5482.

#### DSS colitis and GLP-1–modulating therapies

For induction of acute DSS colitis, between 0.5 and 6% (w/v) DSS (colitis grade, MPbio) was supplemented in drinking water for 5 days. A 2% sucrose was added to DSS solutions to ensure consistent palatability and consumption across all experimental groups. Mice were switched to FDD 7 days before DSS supplementation or maintained on ND during course of experiment. As indicated, FDD mice were intraperitoneally injected with GLP-1 receptor agonist (0.1 μg/g; Exendin-4, AS-24463, AnaSpec) or GLP-1 receptor antagonist (0.4 μg/g; Exendin-9-39, AS-24467, AnaSpec) in 100 μl of phosphate-buffered saline (PBS) every day starting 1 day before start of DSS. In addition, mice were orally gavaged with either 10^10^ CFU *E. coli* Nissle (*EcN*), 10^10^ CFU *E. coli* Nissle (*EcN-plasmid*), or 10^10^ CFU *E. coli* Nissle-Hld_SE_ (*EcN-Hld_SE_*) every day starting 1 day before DSS supplementation. Furthermore, mice received 3% (w/v) sodium butyrate (Sigma-Aldrich) in drinking water starting upon switching to FDD 7 days before DSS supplementation. Notably, tissue samples for RNA sequencing analysis are derived from mice either gavaged with *EcN* (control and GLP-1 receptor agonist groups) or *EcN-Hld_SE_* (*EcN-Hld_SE_* and GLP-1 receptor antagonist groups). Mice were monitored daily for changes in body weight. After 5 days, mice were euthanized for blood and tissue sampling. In addition, FDD mice were orally gavaged with 40 mg DSS per mouse in 200 μl of PBS every day for 4 days to ensure equal uptake of DSS while treatment with GLP-1 receptor agonist.

#### Lamina propria cell isolation and flow cytometry analysis

Leukocyte isolation from colon was performed as previously described (*46*). In brief, large intestine was removed and opened longitudinally. After washing of tissue in PBS, the intestine was cut into 0.5-cm-long pieces and incubated in 5 mM EDTA solution [RPMI, 5% fetal calf serum (FCS)] twice for 20 min at 37°C under agitation of 600 rpm. After washing with PBS, lamina propria cells were isolated by digesting the tissue pieces with collagenase D, deoxyribonuclease (DNase) and dispase (in RPMI, 10% FCS) for 50 min at 37°C under agitation of 1000 rpm. The supernatant of the digest was pelleted, and leukocytes were further purified using 80:40 Percoll gradient. After isolation, cells were analyzed immediately by flow cytometry. For surface staining of fresh cells, cell suspensions were blocked with Fc-blocking solution and stained with antibody mix for 20 min at 4°C. Data acquisition was performed using a five-laser Fortessa flow cytometer (BD Bioscience), and analysis was carried out using FlowJo software (TreeStar).

#### Histologic analysis and immunohistochemistry

The intestines were carefully rinsed with PBS to remove fecal matter. Colon and small intestines were fixed in 10% neutral-buffered formalin for 24 hours and embedded in paraffin. A 4-μm section was used for staining with hematoxylin and eosin (H&E) and Alcian blue (AB) and also for immunohistochemical (IHC) staining for GLP-1 (GLP-1 polyclonal antibody, PA5-79303, Invitrogen). A pathologist assessed and scored the H&E-stained slides for severity of DSS colitis using a method described previously (*47*).

#### Plating for liver CFU

For bacterial extraction from liver tissue, livers were first weighed and then transferred into sterile stainless steel microvials (Biospec, #2007) containing two stainless steel beads (6.35 mm; Biospec, #11079635SS) and capped with silicone rubber caps (Biospec, #2008). The vials were subsequently filled with 500 μl of sterile PBS, and the tubes were placed on ice to prevent bacterial growth. The samples were homogenized using a Mini-BeadBeater-16 (BioSpec) with three cycles of 30 s at 4°C. After extraction, the samples were diluted as needed in PBS, and 100 μl of the diluted solution was plated onto Luria-Bertani agar plates (2% agar). The plates were thoroughly dried and incubated at 37°C overnight. CFU were counted the following day and normalized to tissue weight. Steady-state control mice (ND and FDD without DSS treatment) consistently yielded near-zero CFU counts, confirming that positive signals reflected colitis-induced barrier disruption rather than background contamination.

#### Plasma analyses

GLP-1 and proinflammatory cytokine measurements were performed from plasma samples. After euthanasians, the blood was drawn from mice with needles coated with EDTA to stop coagulation. Blood samples were pelleted by centrifugation for 10 min at 15,000 rpm at 4°C. Supernatant was transferred to a new tube, snap-frozen, and stored at −80°C. GLP-1 enzyme-linked immunosorbent assay (ELISA) (GLP-1 Multispecies ELISA kit, BMS2194, Invitrogen) and LEGENDplex Mouse Inflammation Panel (740446, BioLegend) were performed according to the manufacturer’s protocol.

#### Quantitative PCR

A 3 mm of distal colon was transferred into 2-ml lysin matrix D tubes (6913050, MP Biomedicals) containing 1 ml of TRIzol reagent (15596026, Invitrogen), and the tissue was disrupted by bead beating. RNA was isolated according to the manufacturer’s manual. RNA was subjected to reverse transcription with QScript cDNA SuperMix (QuantaBio). Quantitative polymerase chain reaction (qPCR) was performed using SYBER Green. Samples were analyzed using the ΔΔ*C*t method and normalized to *Gapdh*.

#### Colonic transcriptional analysis

For mRNA sequencing using the Illumina platform, total RNA was first extracted from tissue using TRIzol reagent (15596026, Invitrogen). The extracted RNA was quantified using Bioanalyzer to ensure high-quality RNA (RNA integrity number > 7) for further library preparation. polyadenylate [poly(A)] mRNA was isolated using the Dynabeads mRNA Purification Kit (Thermo Fisher Scientific, #61006), followed by fragmentation with the NEBNext Ultra II RNA Library Prep Kit [New England Biolabs (NEB), #E7770] at 94°C for 8 min. First-strand cDNA synthesis was performed using SuperScript IV Reverse Transcriptase (Thermo Fisher Scientific, #18090050), followed by second-strand cDNA synthesis. After adapter ligation with Illumina TruSeq Adapters (Illumina, #20015960), the cDNA libraries were purified using AMPure XP Beads (Beckman Coulter, #A63881) and amplified using NEBNext High-Fidelity PCR Master Mix (NEB, #M0541). Libraries were validated for quality and size distribution using 2% agarose gel and enriched using size selection beads to eliminate any cDNA below 350 base pairs (bp) and quantified using a Qubit 4 Fluorometer (Thermo Fisher Scientific, #Q33226). Sequencing was performed on an Illumina NextSeq550 platform using paired-end 150-bp reads.

Postsequencing, FastQC (v0.11.9) was used to assess raw read quality, evaluating metrics such as per-base sequence quality (Q30 or higher), gas chromatography content, and potential adapter contamination. Any necessary trimming of low-quality sequences and adapters was carried out using Trim Galore. Reads alignment and transcripts quantification of gene expression were done using Kallisto (v0.46.1), and DESeq2 (v1.30.1) was then used for differential expression analysis, incorporating normalization and variance-stabilizing transformation to account for differences in sequencing depth and library size. Biological replicate consistency was checked using principal components analysis (PCA) to ensure reliable clustering of replicates, and any outliers were flagged for further examination or removal. Functional enrichment analysis was conducted using GSEA and Kyoto Encyclopedia of Genes and Genomes pathway enrichment analysis. GSEA was performed using GSEA v4.3.2 software (Broad Institute Inc., Massachusetts Institute of Technology, and Regents of the University of California).

#### 16S rRNA gene sequencing

##### DNA extraction and purification

Genomic DNA was extracted from fecal samples (one disrupted pellet in 500 μl of PBS) stored at −80°C using a bead-beating approach. Briefly, ~200 μl of 0.1 mm zirconia per glass beads were added to a 96-well deep-well plate. Each well received 300 μl of lysis buffer [50 mM tris-HCl (pH 7.5), 0.2 mM EDTA, and DNA/RNA shield buffer as DNase inhibitor), followed by 100 μl of fecal matter suspension. Plates were centrifuged at 4300*g* for 4 min at 2°C and subjected to bead beating (3 cycles, 2.5 min each) with intermittent cooling on ice. After centrifugation (4300*g*, 5 min, 2°C), 60 μl of lysate was transferred and mixed with Proteinase K solution (50 μg/μl, final concentration) and incubated (65°C for 30 min, followed by 95°C for 30 min). DNA purification was performed using a 1X SPRI bead cleanup protocol, eluting DNA into 27 μl of nuclease-free water (NFW).

##### PCR amplification

The bacterial 16*S* ribosomal RNA (rRNA) gene V4 region (~253 bp) was amplified using modified EMP primers: forward primer (515f): 5′-GTGYCAGCMGCCGCGGTAA-3′ and reverse primer (806rB): 5′-GGACTACNVGGGTWTCTAAT-3′. Primers included NexteraXT adapters for indexing. PCR reactions (20 μl) contained 2 μl of template DNA, 10 μl of 2X NEBNext HiFi HotStart Master Mix, 0.2 μl of 10X SYBR Green, 5.8 μl of NFW, and 2 μl of primer mix. Reactions underwent qPCR cycling stopped in the exponential phase (~13 to 14 cycles).

##### Library preparation and sequencing

PCR products were pooled based on qPCR fluorescence intensities, cleaned using a 2X SPRI bead protocol, and eluted in 45 μl of NFW. Pooled samples underwent size selection via 2% E-Gel electrophoresis, and target bands were excised and purified with Monarch gel extraction kits. Final purified libraries were quantified using a Qubit fluorometer.

##### Sequencing and bioinformatics

Analysis Amplicon sequencing was performed using an Illumina MiSeq system (300 V2 kit). Raw sequencing reads were demultiplexed and analyzed using the USEARCH pipeline (version 10). Reads were merged with a maximum of 10 allowed mismatches, and primer sequences were identified, filtered, and trimmed. Reads were oriented using the SILVA 138 database, followed by quality filtering based on expected errors (maximum expected error ≤ 1). Sequences containing ambiguous bases (Ns) were discarded. Primer identification and trimming were performed using custom scripts to ensure accurate alignment and removal of mis-primed sequences. The dereplication was performed to identify unique sequences, which were then denoised using the UNOISE3 algorithm to generate zero-radius operational taxonomic units (ZOTUs/ASVs). An OTU table was constructed using sequences postfiltering and primer trimming. The differential abundance analysis was conducted using DESeq2. PCA was performed using normalized ASV data to assess microbial community variation. Taxonomic classification was performed using a naive Bayes classifier trained on the SILVA 138 database specific to the V4 region. All bioinformatics tools and statistical analyses used have been previously described (*48*–*50*). Beta-diversity was assessed by principal coordinates analysis on Aitchison distances (Euclidean on centered log ratio-transformed ASV counts, pseudocount = 1) with significance tested by permutational multivariate analysis of variance (PERMANOVA) (999 permutations), and alpha diversity was estimated as Chao1 richness in phyloseq, without rarefaction and group comparisons across, was performed by two-sided Wilcox test.

#### Engineering EcN-Hld_SE_/EcN-plasmid

We used *E. coli* Nissle as a bacterial chassis to develop a probiotic strain that secretes the Hld_SE_ peptide from *S. epidermidis* (*28*) in vivo. To determine the optimal secretion system for producing the most effective phenotype, we explored two different signal peptides: PelBss, commonly used for heterologous protein secretion in *E. coli*, and the NSP4 peptide, which has recently been shown to improve secretion efficiency and outperform PelBss. To avoid potential toxicity from the Hld_SE_ peptide, we cloned the construct into the pCOLADuet-1 vector (Novagen, #71406), a medium copy number plasmid optimized for protein expression. We synthesized two gBlocks (Integrated DNA Technologies, Inc.), each containing a synthetic strong promoter (J23100, Table 2, bold text), a signal peptide (Table 2, underlined text), and the Hld_SE_ sequence (Table 2, italic text). These constructs were cloned into the pCOLADuet-1 vector using the forward primer: CTTACATTAATTGCGTTGCGTTGACGGCTAGCTCAGTC and the reverse primer: GATACCGGTAGGCTGCATTTTTTTAAATTTGTTCACGGTATCAAT. In addition, to isolate the efficacy of the secreted peptide, we developed a control plasmid lacking the peptide’s coding region and secretion tag fusion. Using NSP4-Hld_SE_ as a template, primers were developed to excise this portion and leave the rest of the plasmid intact (Table 2). The plasmids were assembled using NEBuilder HiFi DNA Assembly Master Mix (NEB, #E2621) and transformed into *E. coli* Nissle, with transformants selected on kanamycin-containing media. All constructs were sequenced verified: pCOLAduet-NSP4-Hld_SE_ plasmid sequence (TTGACGGCTAGCTCAGTCCTAGGTACAGTGCTAGCTACTAGAGAAAGAGGAGAAATACTAGATGAAAAAGATTACCGCTGCTGCTGGTCTGCTGCTCCTCGCTGCCCAGCCGGCGATGGCGATGGCGGCGGATATTATTAGCACCATTGGCGATCTGGTGAAATGGATTATTGATACCGTGAACAAATTTAAAAAAATGCAGCCTACCGGTATCCTAGGCTGCTGCCACCGCTGAGCAATAACTAGCATAACCCCTTGGGGCCTCTAAACGGGTCTTGAGGGGTTTTTTGCTGAAACCTCAGGCATTTGAGAAGCACACGGTCACACTGCTTCCGGTAGTCAATAAACCGGTAAACCAGCAATAGACATAAGCGGCTATTTAACGACCCTGCCCTGAACCGACGACAAGCTGACGACCGGGTCTCCGCAAGTGGCACTTTTCGGGGAAATGTGCGCGGAACCCCTATTTGTTTATTTTTCTAAATACATTCAAATATGTATCCGCTCATGAATTAATTCTTAGAAAAACTCATCGAGCATCAAATGAAACTGCAATTTATTCATATCAGGATTATCAATACCATATTTTTGAAAAAGCCGTTTCTGTAATGAAGGAGAAAACTCACCGAGGCAGTTCCATAGGATGGCAAGATCCTGGTATCGGTCTGCGATTCCGACTCGTCCAACATCAATACAACCTATTAATTTCCCCTCGTCAAAAATAAGGTTATCAAGTGAGAAATCACCATGAGTGACGACTGAATCCGGTGAGAATGGCAAAAGTTTATGCATTTCTTTCCAGACTTGTTCAACAGGCCAGCCATTACGCTCGTCATCAAAATCACTCGCATCAACCAAACCGTTATTCATTCGTGATTGCGCCTGAGCGAGACGAAATACGCGGTCGCTGTTAAAAGGACAATTACAAACAGGAATCGAATGCAACCGGCGCAGGAACACTGCCAGCGCATCAACAATATTTTCACCTGAATCAGGATATTCTTCTAATACCTGGAATGCTGTTTTCCCGGGGATCGCAGTGGTGAGTAACCATGCATCATCAGGAGTACGGATAAAATGCTTGATGGTCGGAAGAGGCATAAATTCCGTCAGCCAGTTTAGTCTGACCATCTCATCTGTAACATCATTGGCAACGCTACCTTTGCCATGTTTCAGAAACAACTCTGGCGCATCGGGCTTCCCATACAATCGATAGATTGTCGCACCTGATTGCCCGACATTATCGCGAGCCCATTTATACCCATATAAATCAGCATCCATGTTGGAATTTAATCGCGGCCTAGAGCAAGACGTTTCCCGTTGAATATGGCTCATACTCTTCCTTTTTCAATATTATTGAAGCATTTATCAGGGTTATTGTCTCATGAGCGGATACATATTTGAATGTATTTAGAAAAATAAACAAATAGGCATGCTAGCGCAGAAACGTCCTAGAAGATGCCAGGAGGATACTTAGCAGAGAGACAATAAGGCCGGAGCGAAGCCGTTTTTCCATAGGCTCCGCCCCCCTGACGAACATCACGAAATCTGACGCTCAAATCAGTGGTGGCGAAACCCGACAGGACTATAAAGATACCAGGCGTTTCCCCCTGATGGCTCCCTCTTGCGCTCTCCTGTTCCCGTCCTGCGGCGTCCGTGTTGTGGTGGAGGCTTTACCCAAATCACCACGTCCCGTTCCGTGTAGACAGTTCGCTCCAAGCTGGGCTGTGTGCAAGAACCCCCCGTTCAGCCCGACTGCTGCGCCTTATCCGGTAACTATCATCTTGAGTCCAACCCGGAAAGACACGACAAAACGCCACTGGCAGCAGCCATTGGTAACTGAGAATTAGTGGATTTAGATATCGAGAGTCTTGAAGTGGTGGCCTAACAGAGGCTACACTGAAAGGACAGTATTTGGTATCTGCGCTCCACTAAAGCCAGTTACCAGGTTAAGCAGTTCCCCAACTGACTTAACCTTCGATCAAACCGCCTCCCCAGGCGGTTTTTTCGTTTACAGAGCAGGAGATTACGACGATCGTAAAAGGATCTCAAGAAGATCCTTTACGGATTCCCGACACCATCACTCTAGATTTCAGTGCAATTTATCTCTTCAAATGTAGCACCTGAAGTCAGCCCCATACGATATAAGTTGTAATTCTCATGTTAGTCATGCCCCGCGCCCACCGGAAGGAGCTGACTGGGTTGAAGGCTCTCAAGGGCATCGGTCGAGATCCCGGTGCCTAATGAGTGAGCTAACTTACATTAATTGCGTTGCG).

**Table 2.** Components of synthetic gBlocks and control constructs used for Hld_SE_ expression.

*pCOLA_NSP4_ Hld_SE_*	**TTGACGGCTAGCTCAGTCCTAGGTACAGTGCTAGCTACTAGAGAAAGAGGAGAAATACTGATGAAAAAGATTACCGCTGCTGCTGGTCTGCTGCTCCTCGCTGCCCAGCCGGCGATGGCG** ***ATGGCGGCGGATATTATTAGCACCATTGGCGATCTGGTGAAATGGATTATTGATACCGTGAACAAATTTAAAAAA***
*pCOLA_PELB5_ Hld_SE_*	**TTGACGGCTAGCTCAGTCCTAGGTACAGTGCTAGC**TACTAGAGAAAGAGGAGAAATACTAGATGAAGTACCTGCTGCCGACCGCGGCGGCGGGTCTGCTGCTGCTGGCGGCGCAGCCGGCGATGGCGGACGATGACGATGAC*ATGGCGGCGGATATTATTAGCACCATTGGCGATCTGGTGAAATGGATTATTGATACCGTGAACAAATTTAAAAAA*
*pCOLA_empty_F*	**GAAATACTAGA**TGCAGCCTACCGGTATC
*pCOLA_empty_R*	**TAGGCTGCAT**CTAGTATTTCTCCTCTTTCTCTAG

### Statistical analysis

Statistical significance was determined by unpaired *t* test with Welch’s correction or other methods as noted on figure legends. *P* values are represented on figures as follows: ns, not significant, *P < 0.05, **P < 0.01, ***P < 0.005, ****P < 0.001, and *****P < 0.0005. Error bars on all figures represent SEM, if not otherwise indicated. Statistical analysis was performed using GraphPad Prism version 10 for macOS (GraphPad Software).
