# Supplementary material for: Engineered probiotic restores GLP-1 signaling to ameliorate fiber-deficiency exacerbated colitis
Source: Sci Adv. 2025 Nov 7;11(45):eadx6869. doi: 10.1126/sciadv.adx6869 (PMC12594200; doi:10.1126/sciadv.adx6869)
Supplement: Supplementary file 1 — Figs. S1 to S6 [file sciadv.adx6869_sm.pdf]

Science Advances
AAAS

Supplementary Materials for

Engineered probiotic restores GLP-1 signaling to ameliorate fiber-deficiency exacerbated colitis

Leonie Brockmann *et al.*

Corresponding author: Harris H. Wang, harris.wang@columbia.edu

Sci. Adv. **11**, eadx6869 (2025)
DOI: 10.1126/sciadv.adx6869

This PDF file includes:

Figs. S1 to S6

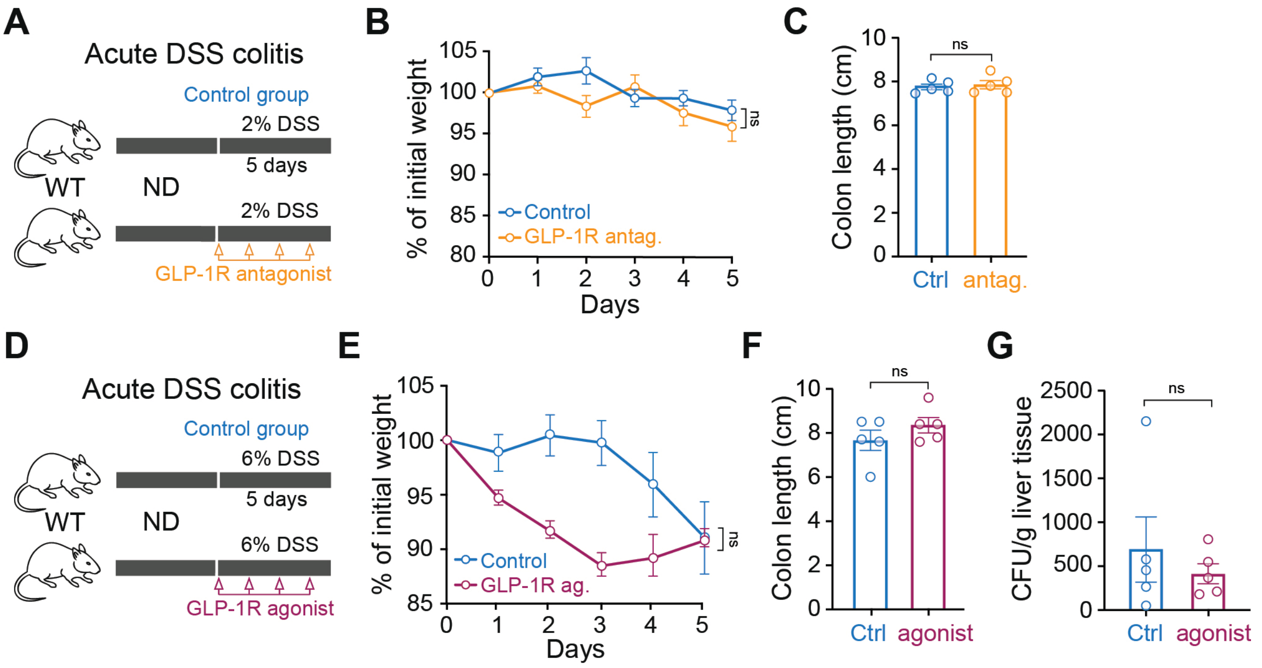

Supplemental Figure S1. GLP-1 receptor antagonism does not exacerbate mild colitis, while GLP-1 receptor agonism provides only modest benefit in severe acute colitis, related to Figure 1.

(A) Experimental schematic of ND mice under 2% DSS colitis condition with additional treatment with GLP-1 receptor antagonist (Exendin-9-39).

(B) Weight development of ND mice during five days of DSS colitis with or without treatment with GLP-1 receptor antagonist. One experiments, n= 5 mice/group.

(C) Colon length of ND mice after five days of DSS colitis with or without treatment with GLP-1 receptor antagonist. One experiments, n= 5 mice/group.

(D) Experimental schematic of ND mice under 6% DSS colitis condition with additional treatment with GLP-1 receptor agonist (Exendin-4).

(E) Weight development of ND mice during five days of DSS colitis with or without treatment with GLP-1 receptor agonist. One experiments, n= 5 mice/group.

(F) Colon length of ND mice after five days of DSS colitis with or without treatment with GLP-1 receptor agonist. One experiments, n= 5 mice/group.

(G) Colony forming units (CFU) in liver of ND mice after five days of DSS colitis with or without treatment with GLP-1 receptor agonist. One experiments, n= 5 mice/group.

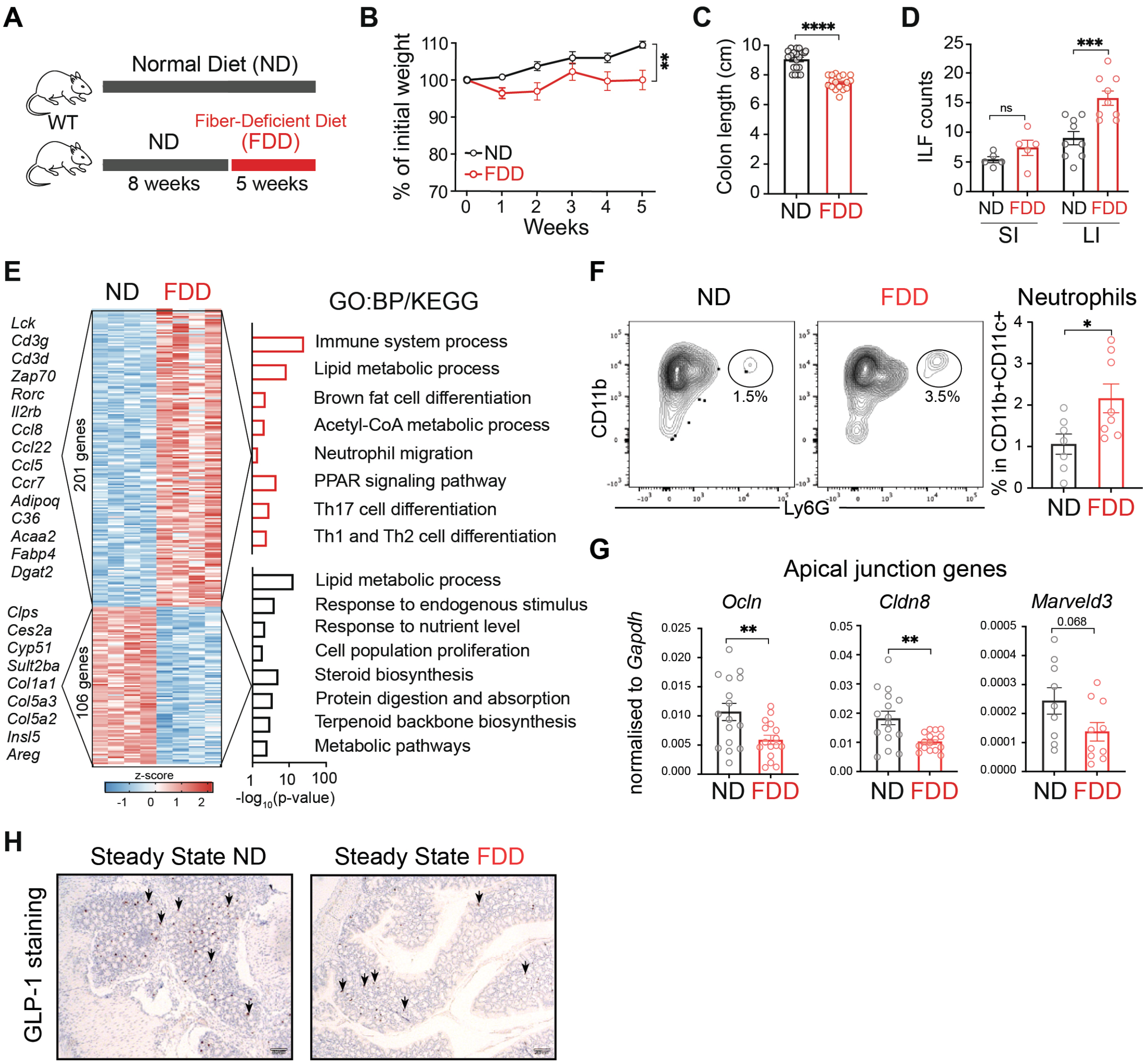

Supplemental Figure S2. Fiber deficient diet causes disruption of immune and enteroendocrine homeostasis, related to Figure 2.

(A) Experiment schematic of dietary intervention under steady state condition.

(B) Weight development of mice fed normal diet (ND) or fiber-deficient diet (FDD) for five weeks. Cumulative of two independent experiment, n= 6-8 mice/group.

(C) Colon length after feeding of ND or FDD for five weeks. Cumulative of four independent experiment, n= 17-20 mice/group.

(D) Isolated lymphoid follicle (ILF) count in the SI and LI of ND and FDD mice after 5 weeks of dietary intervention. Two experiment, n= 5-9 mice/group.

(E) Heatmap of differentially expressed genes (DEGs) from RNA-sequencing of colon from mice fed ND or FDD for five weeks. One experiment, n= 4 mice/group, top KEGG and GO:BP pathways in over-representation analysis on the right and example DEGs on the left.

(F) CD11b and Ly6G expression (neutrophils) in myeloid cells ($CD11c^{+}$ $CD11b^{+}$ $TCRb^{-}$ $B220^{-}$) in colon of mice fed ND or FDD for five weeks. Cumulative of two independent experiments, n= 7-8 mice/group.

(G) Quantitative PCR for selected apical junction genes from colon of mice fed ND or FDD for five weeks. Cumulative of three independent experiments, n= 10-16 mice/group.

(H) Representative GLP-1 staining in colon of mice fed ND or FDD for five weeks.

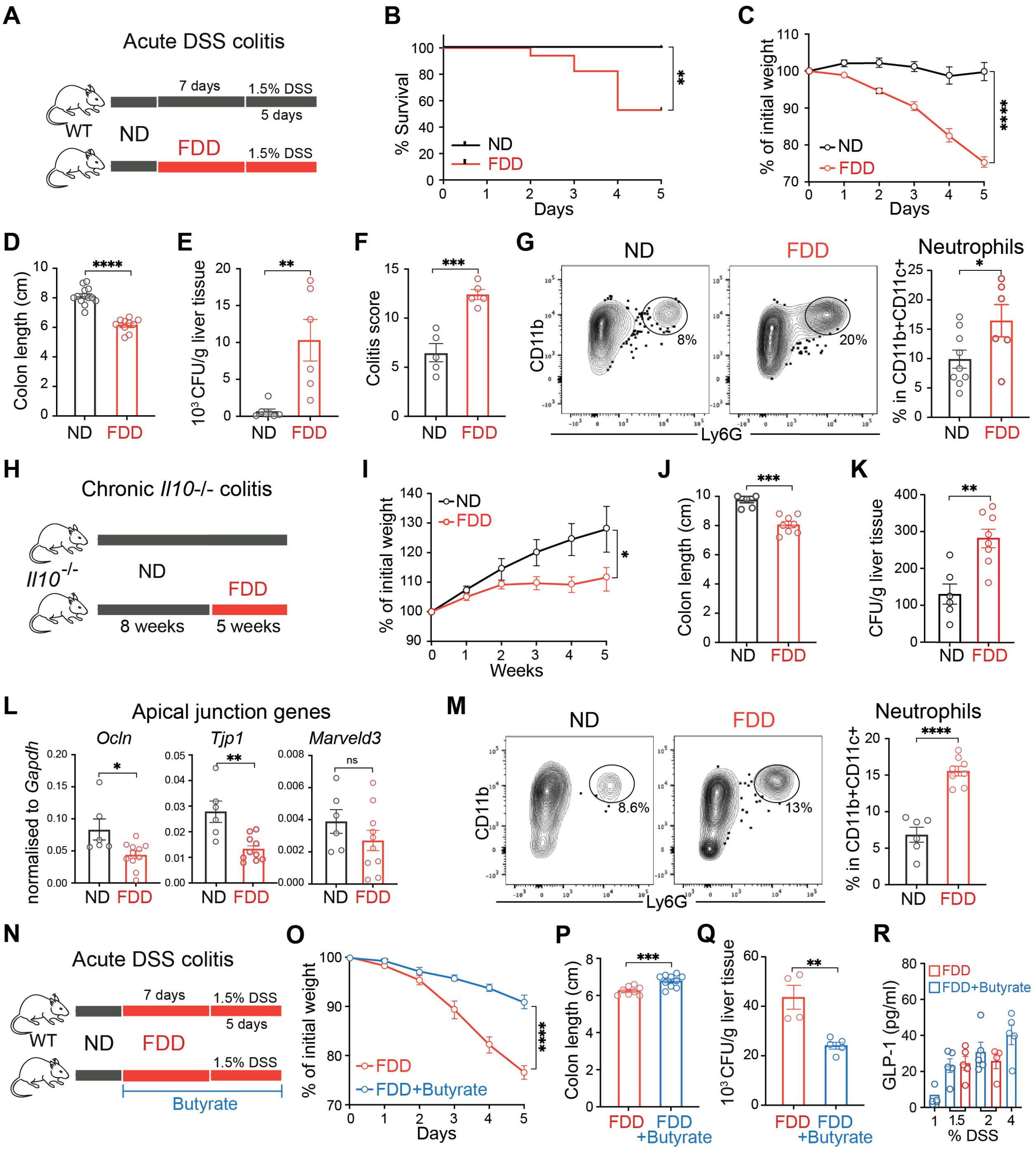

Supplemental Figure S3. Dietary fiber deficiency causes severe colitis susceptibility, related to Figure 2.

(A) Experiment schematic of dietary intervention under DSS colitis conditions.

(B) Survival curve of mice fed FDD or ND for seven days prior to five days of DSS colitis. Cumulative of three independent experiments, n= 13-17 mice/group.

(C) Weight development of FDD or ND mice during five days of DSS colitis. Cumulative of three independent experiments, n= 13-17 mice/group.

(D) Colon length of FDD or ND mice after five days of DSS colitis. Cumulative of three independent experiments, n= 10-13 mice/group.
(E) Colony forming units (CFU) in liver of FDD or ND mice after five days of DSS colitis. Cumulative of two independent experiments, n= 6-7 mice/group.
(F) Colitis score based on assessment of H&E staining of colon from FDD or ND mice after five days of DSS colitis. One experiments, n= 4 mice/group.
(G) CD11b and Ly6G expression (neutrophils) in myeloid cells ($CD11c^+$ $CD11b^+$ $TCRb^-$ $B220^-$) in colon of mice ND or FDD mice after five days of DSS colitis. Cumulative of two independent experiments, n= 6-9 mice/group.
(H) Experiment schematic of dietary intervention in *Il10*$^{-/-}$ mice.
(I) Weight development of *Il10*$^{-/-}$ mice fed ND or FDD for five weeks. Cumulative of two independent experiment, n= 6-8 mice/group.
(J) Colon length of *Il10*$^{-/-}$ mice fed ND or FDD for five weeks. Cumulative of two independent experiment, n= 6-8 mice/group.
(K) CFU in liver of *Il10*$^{-/-}$ mice fed ND or FDD for five weeks. Cumulative of three independent experiment, n= 6-11 mice/group.
(L) Quantitative PCR for selected apical junction genes from colon of *Il10*$^{-/-}$ mice fed ND or FDD for five weeks. Cumulative of two independent experiments, n= 6-10 mice/group.
(M) CD11b and Ly6G expression (neutrophils) in myeloid cells ($CD11c^+$ $CD11b^+$ $TCRb^-$ $B220^-$) in colon of mice ND- or FDD fed *Il10*$^{-/-}$ mice after five weeks of dietary intervention. Cumulative of two independent experiments, n= 6-8 mice/group.
(N) Experiment schematic of dietary intervention under DSS colitis conditions with additional supplementation of butyrate.
(O) Weight development of FDD mice during five days of DSS colitis with or without butyrate supplementation. Cumulative of two independent experiments, n= 10 mice/group.
(P) Colon length of FDD mice after five days of DSS colitis with or without butyrate supplementation. Cumulative of two independent experiments, n= 8-10 mice/group.
(Q) CFU in liver of FDD mice after five days of DSS colitis with or without butyrate supplementation. One independent experiments, n= 4-5 mice/group.
(R) Plasma GLP-1 level of FDD mice after five days of DSS colitis with or without butyrate supplementation. Cumulative of two independent experiments, n= 4-5 mice/group.

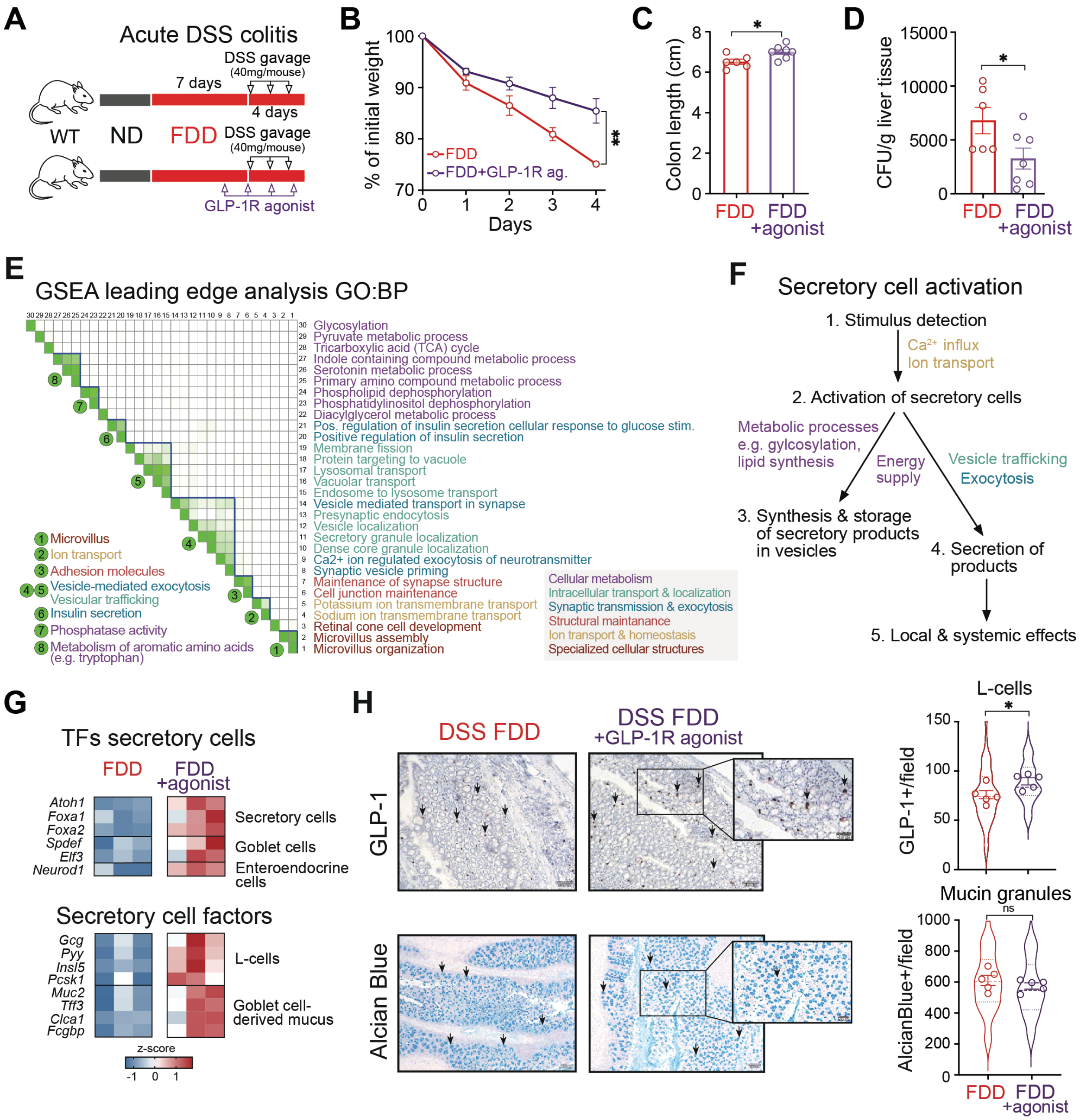

Supplemental Figure S4. GLP-1 receptor agonists enhance gene expression of secretory cell functions in colitis, related to Figure 3.

(A) Experiment schematic of DSS colitis conditions with GLP-1 receptor agonist (Exendin-4) treatment in FDD mice. Mice were orally gavaged with 40 mg DSS/mouse/day in 200 µl PBS.

(B) Weight development of FDD mice during four days of DSS colitis with or without GLP-1 receptor agonist treatment. Cumulative of two independent experiments, n= 8 mice/group.

(C) Colon length of FDD mice after four days of DSS colitis with or without GLP-1 receptor agonist treatment. Cumulative of two independent experiments, n= 6-7 mice/group.

(D) CFU in liver of FDD mice after four days of DSS colitis with or without GLP-1 receptor agonist treatment. Cumulative of two independent experiments, n= 6-7 mice/group.
(E) Leading-edge analysis from top 30 most enriched GO:BP pathways in GSEA from RNA-sequencing samples in Figure 3G.
(F) Schematic of intestinal secretory cell activation and secretion.
(G) Heatmap of selected upregulated transcription factors and upregulated secretory cell linage related genes from RNA-sequencing samples in Figure 3G.
(H) Number of L-cells based on GLP-1 staining and mucin granules based on Alcian Blue staining in colon of mice fed FDD with or without GLP-1 receptor agonist treatment during DSS colitis. Data are shown as the average per biological replicate (n = 5 mice/group; 10 fields/colon), overlaid on a violin plot representing all imaged fields. Arrows indicate examples of GLP-1– and Alcian Blue–positive signals. One experiment.

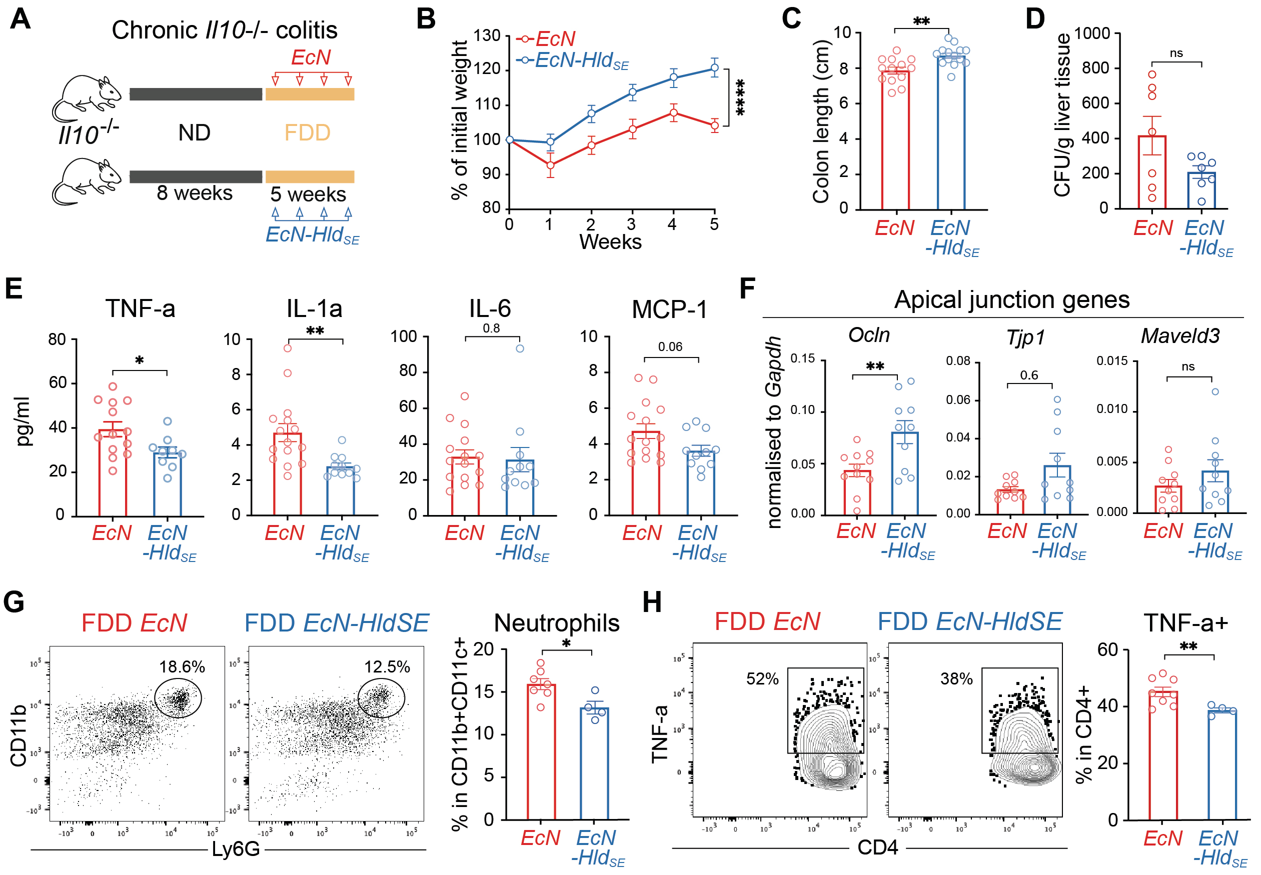

Supplemental Figure S5. An engineered probiotic promotes intestinal health in dysbiotic *Il10*$^{-/-}$ mice, related to Figure 4.

(A) Experiment schematic of dietary intervention in *Il10*$^{-/-}$ mice with additional gavage of 10^{10} CFU/mouse *EcN* or *EcN-Hld*$_{SE}$ every other day for five weeks.

(B) Weight development of FDD *Il10*$^{-/-}$ mice with additional gavage of 10^{10} CFU/mouse *EcN* or *EcN-Hld*$_{SE}$ every other day for five weeks. Cumulative of three independent experiments, n= 13-14 mice/group.

(C) Colon length of FDD *Il10*$^{-/-}$ mice with additional gavage of 10^{10} CFU/mouse *EcN* or *EcN-Hld*$_{SE}$ every other day for five weeks. Cumulative of three independent experiments, n= 13-14 mice/group.

(D) CFU in liver of FDD *Il10*$^{-/-}$ mice with additional gavage of 10^{10} CFU/mouse *EcN* or *EcN-Hld*$_{SE}$ every other day for five weeks. Cumulative of two independent experiments, n= 7-11 mice/group.

(E) Concentration of plasma TNF-α, IL-1β, IL-6 and MCP-1 of FDD *Il10*$^{-/-}$ mice with additional gavage of 10^{10} CFU/mouse *EcN* or *EcN-Hld*$_{SE}$ every other day for five weeks. Cumulative of three independent experiments, n= 11-14 mice/group.

(F) Quantitative PCR for selected apical junction genes from colon of *Il10*$^{-/-}$ mice fed FDD with additional gavage of 10^{10} CFU/mouse *EcN* or *EcN-Hld*$_{SE}$ every other day for five weeks. Cumulative of two independent experiments, n= 10-11 mice/group.

(G) CD11b and Ly6G expression (neutrophils) in myeloid cells (CD11c^{+} CD11b^{+} TCRb^{-} B220^{-}) in colon of FDD fed *Il10*$^{-/-}$ mice with additional gavage of 10^{10} CFU/mouse *EcN*

or *EcN-Hld*$_{SE}$ every other day for five weeks. Cumulative of two independent experiments, n= 4-7 mice/group.

(H) TNF-α expression (inflammatory T cells) in $CD4^+$ T cells ($CD4^+$ $TCRb^+$) in colon of FDD fed *Il10*$^{-/-}$ mice with additional gavage of 10^{10} CFU/mouse *EcN* or *EcN-Hld*$_{SE}$ every other day for five weeks. Cumulative of two independent experiments, n= 4-8 mice/group.

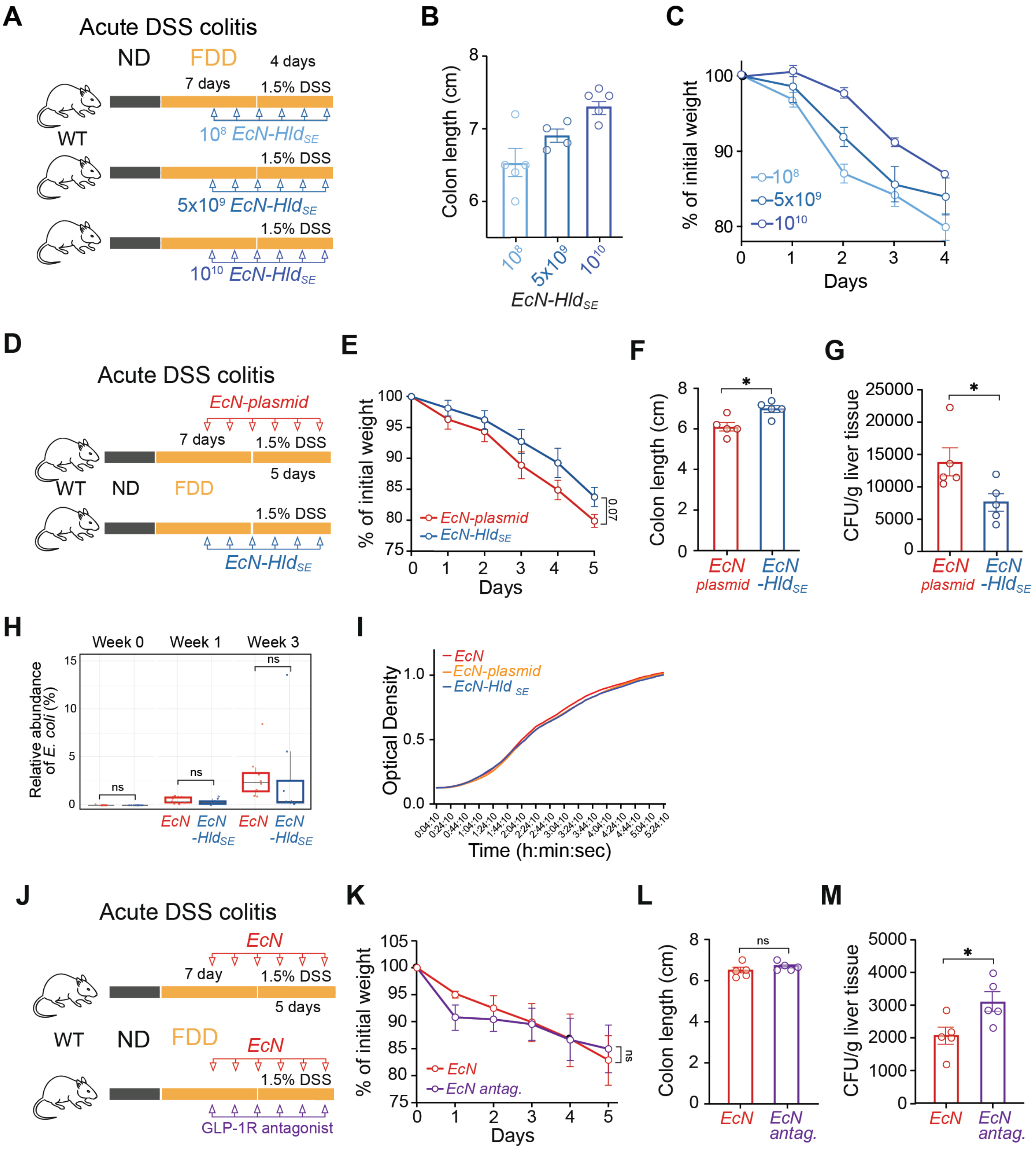

Supplemental Figure S6. Characterization of engineered probiotic dose effect, *EcN-plasmid* control strain, and effect of GLP-1 antagonism on *EcN*-treated FDD mice, related to Figure 5.

(A) Experimental schematic of FDD mice under DSS colitis condition with additional gavage of indicated CFU/mouse *EcN-Hld*$_{SE}$ every day starting two days prior to DSS supplementation.

(B) Colon length of FDD mice after five days of DSS colitis with additional gavage of indicated CFU/mouse *EcN-Hld$_{SE}$* every day. One experiment, n= 4-5 mice/group.
(C) Weight development of FDD mice during five days of DSS colitis with additional gavage of indicated CFU/mouse *EcN-Hld$_{SE}$* every day. One experiment, n= 4-5 mice/group. (A)
(D) Experiment schematic of DSS colitis conditions with either *EcN-plasmid* or *EcN-Hld$_{SE}$* treatment in FDD mice.
(E) Weight development of FDD mice during five days of DSS colitis with either *EcN-plasmid* or *EcN-Hld$_{SE}$* treatment. One experiments, n= 5 mice/group.
(F) Colon length of FDD mice after five days of DSS colitis with either *EcN-plasmid* or *EcN-Hld$_{SE}$* treatment. One experiments, n= 5 mice/group.
(G) CFU in liver of FDD mice after five days of DSS colitis with either *EcN-plasmid* or *EcN-HldS$_{E}$* treatment. One experiments, n= 5 mice/group.
(H) Relative abundance of *E.coli* in 16S rRNA-sequencing data from Figure 4F.
(I) Optical Density (OD) over time of sub-cultured *EcN*, *EcN-plasmid* and *EcN-Hld$_{SE}$*.
(J) Experiment schematic of DSS colitis conditions with *EcN* treatment with or without injection of GLP-1 receptor antagonist (Exendin-9-39) in FDD mice.
(K) Weight development of FDD mice during five days of DSS colitis with *EcN* treatment with or without injection of GLP-1 receptor antagonist. One experiments, n= 5 mice/group.
(L) Colon length of FDD mice after five days of DSS colitis with *EcN* treatment with or without injection of GLP-1 receptor antagonist. One experiments, n= 5 mice/group.
(M) CFU in liver of FDD mice after five days of DSS colitis with *EcN* treatment with or without injection of GLP-1 receptor antagonist. One experiments, n= 5 mice/group.
